# Supplementary figures and images for: Integrating human and ecological dimensions: The importance of stakeholders’ perceptions and participation on the performance of fisheries co-management in Chile
Source: PLoS One. 2021 Aug 11;16(8):e0254727. doi: 10.1371/journal.pone.0254727 (PMC8357100; doi:10.1371/journal.pone.0254727)

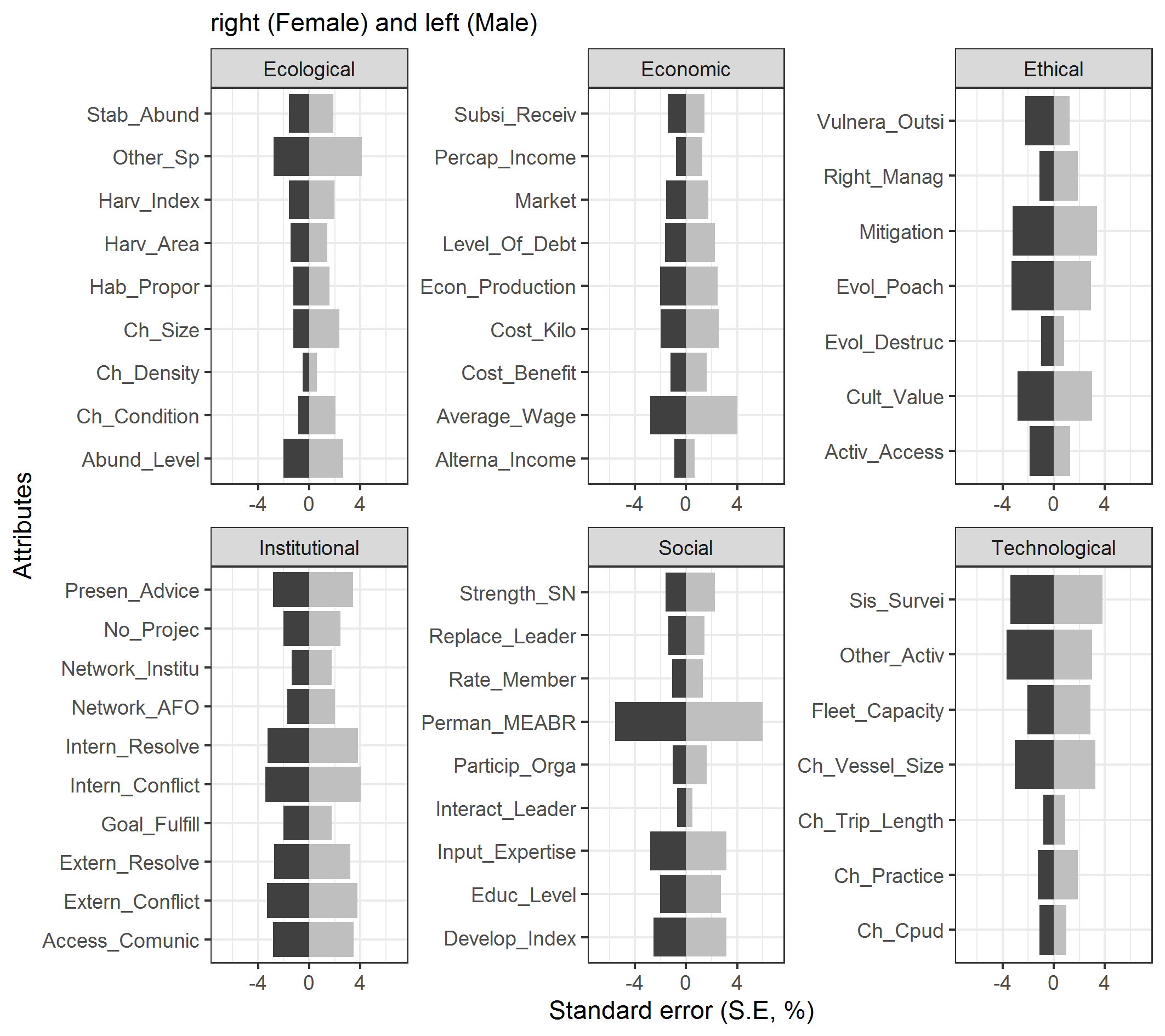

Supplement: S1 Fig — (TIF) [file pone.0254727.s001.tif]

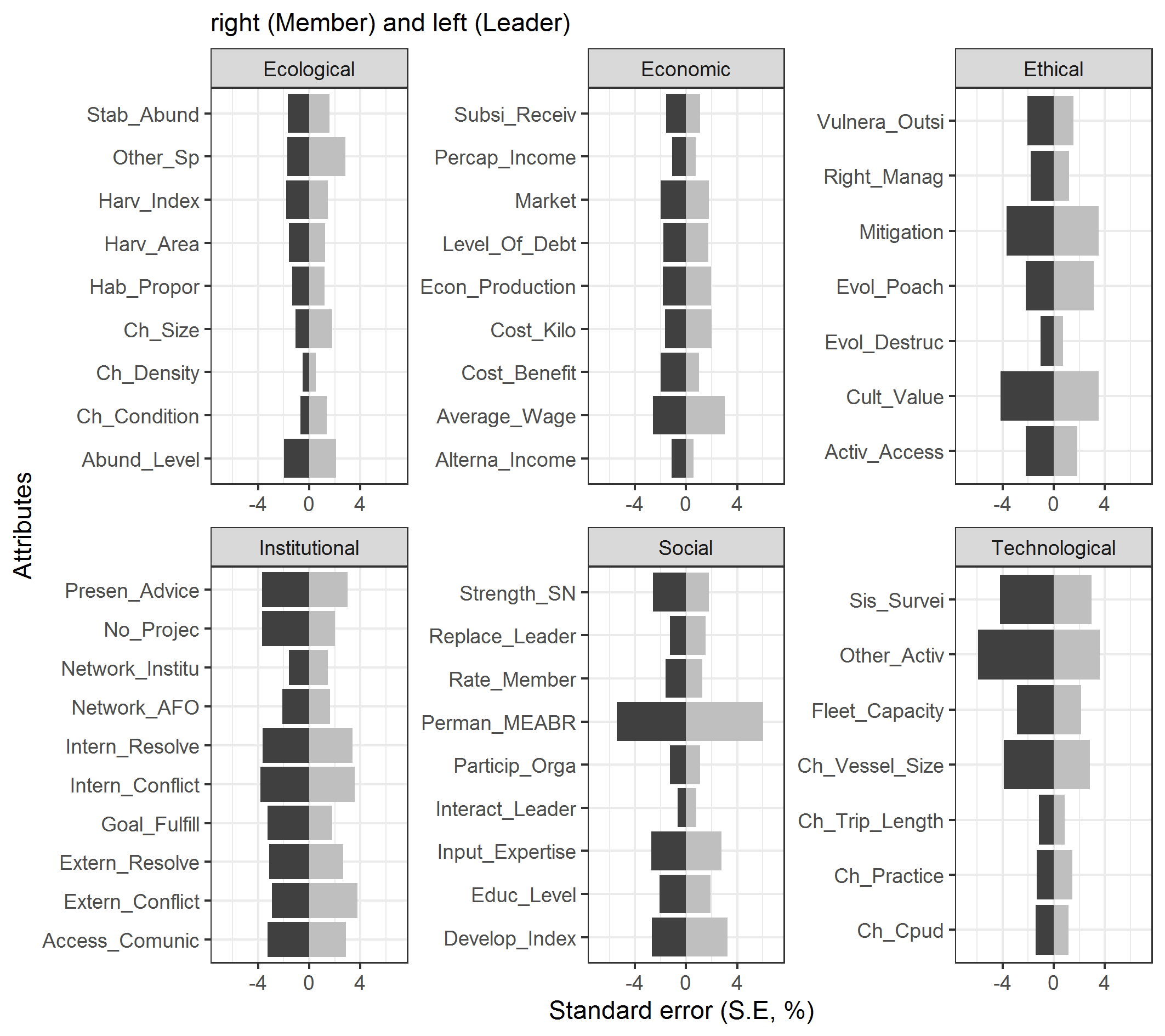

Supplement: S2 Fig — (TIF) [file pone.0254727.s002.tif]

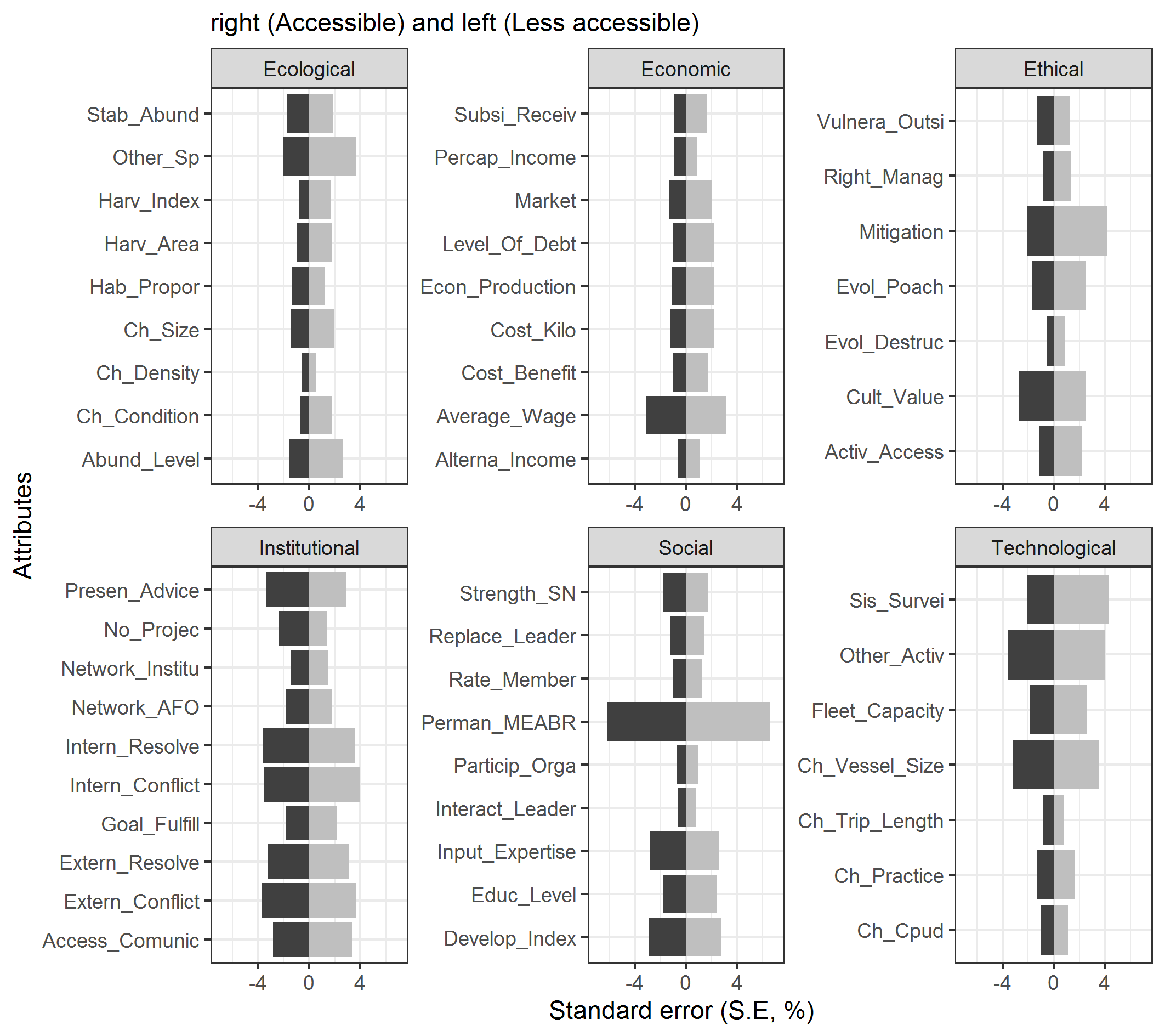

Supplement: S3 Fig — (TIF) [file pone.0254727.s003.tif]
