# Supplementary material for: Integrating human and ecological dimensions: The importance of stakeholders’ perceptions and participation on the performance of fisheries co-management in Chile
Source: PLoS One. 2021 Aug 11;16(8):e0254727. doi: 10.1371/journal.pone.0254727 (PMC8357100; doi:10.1371/journal.pone.0254727)
Supplement: S1 Table — (PDF) [file pone.0254727.s004.pdf]

S1 Table. Description of the four zones in the Biobio Region identified in this study.

Zone	Fishing cove	Geographic location	Township	MEABR name	MEABR Code	Distance from fishing cove to MEABR (km)	Grouped by km
GULF	Laraquete	-37.16623S; -73.186595W	ARAUCO	1. Laraquete	golc	1.494	>0.5
	Punta Lavapie	-37.14921S; -73.579639W	ARAUCO	2. Punta Lavapie	golb	0.434	<0.5
	Maule	-37.00779S; -73.185686W	CORONEL	3. Maule	gola	0.226	<0.5
	Pueblo Hundido	-37.07316S; -73.155369W	LOTA	4. Pueblo Hundido	gold	0.356	<0.5
BAY	Candelaria	-36.62879S; -73.091191W	TALCAHUANO	5. Candelaria-Canteras	bahb	0.383	<0.5
	San Vicente	-36.72561S; -73.132501W	TALCAHUANO	6. San Vicente	baha	3.298	>0.5
	Coliumo	-36.52984S; -72.958528W	TOME	7. Rari	bahc	0.379	<0.5
COAST	Yani	-37.36453S; -73.661106W	ARAUCO	8. Puerto Yana	bcog	1.388	>0.5
	Rumena	-37.17574S; -73.614255W	ARAUCO	9. Punta Raimenco	bcob	2.841	>0.5
	Rumena	-37.17574S; -73.614255W	ARAUCO	10. Bajo Rumena	bcoc	5.423	>0.5
	Rumena	-37.17574S; -73.614255W	ARAUCO	11. Rumena	bcod	0.926	>0.5
	Los Piures	-37.23501S; -73.653844W	ARAUCO	12. Los Piures	bcoe	0.675	>0.5
	Dichato	-36.54764S; -72.944433W	TOME	13. Dichato	bcoa	4.032	>0.5
	Taucu	-36.18039S; -72.814383W	COBQUECURA[†]	14. Cobquecura Sector A	bcof	1.670	>0.5
ISLAND	Pto. Norte (Sta. Ma. Island)	-36.98283S; -73.527800W	CORONEL	15. Pueblo Norte Sector A	insc	1.765	>0.5
	Pto. Norte (Sta. Ma. Island)	-36.98283S; -73.527800W	CORONEL	16. Punta Cadena[ψ]	insd	35.570	>0.5
	Pto. Sur (Sta. Ma. Island)	-37.04503S; -73.51151W	CORONEL	17. Los Partidos	insa	3.440	>0.5
	Pto. Sur (Sta. Ma. Island)	-37.04503S; -73.51151W	CORONEL	18. Puerto Sur	insb	3.440	>0.5
	La Hacienda (Mocha Island)	-38.38027S; -73.96056W	LEBU	19. Weste Isla Mocha	inse	7.451	>0.5
	Islote del Trabajo (Mocha Island)	-38.38027S; -73.96056W	LEBU	20. Quechol	insf	7.451	>0.5
	Islote del Trabajo (Mocha Island)	-38.38027S; -73.96056W	LEBU	21. Quechol Sur	insg	6.878	>0.5

MEABR, management and exploitation area of benthic resources

[†]Until 2017, this township belonged to Biobio Region. Now, it corresponds to Ñuble Region.

[ψ]Despite the management area is in the insular zone; its fishing cove is based in Lo Rojas zone, which corresponds to a continental area, because not all members.
