# Supplementary material for: Integrating human and ecological dimensions: The importance of stakeholders’ perceptions and participation on the performance of fisheries co-management in Chile
Source: PLoS One. 2021 Aug 11;16(8):e0254727. doi: 10.1371/journal.pone.0254727 (PMC8357100; doi:10.1371/journal.pone.0254727)
Supplement: S2 Table — (PDF) [file pone.0254727.s005.pdf]

S2 Table. Characteristics of each Management and Exploitation Area of Benthic Resources (MEABR) in the Biobio Region.

MEABR name	MEABR Code	Number of fishers	Total area (ha)	Year of MEABR application by AFO	Year of first monitoring study	Total number of monitoring study[ψ]	Target specie[*]
1. Laraquete	golc	42	70.71	1998	2011	3	Chilean abalone
2. Punta Lavapie	golb	27	68.45	1997	2006	10	Chilean abalone
3. Maule	gola	27	59.9	1998	2002	13	Chilean abalone
4. Pueblo Hundido	gold	72	14.13	2003	2016	2	Gigartina seaweed
5. Candelaria-Cantera	bahb	61	73.87	1997	2001	15	Chilean abalone
6. San Vicente	baha	34	91.66	1997	2001	13	Chilean abalone
7. Rari	bahc	33	0.82	2004	2006	8	Chicorea seaweed
8. Puerto Yana	bcog	36	112.5	1997	2001	12	Chilean abalone
9. Punta Raimenco[ϕ]	bcob	40	54.78	1997	2003	10	Chilean abalone
10. Bajo Rumena[ϕ]	bcoc		11.5	1997	2003	10	Chilean abalone
11. Rumena	bcod	72	74.48	1997	2001	14	Chilean abalone
12. Los Piures	bcoe	34	271.64	1997	2002	11	Chilean abalone
13. Dichato	bcoa	48	197.58	1997	2000	14	Chilean abalone
14. Cobquecura Sector A	bcof	28	452.5	2001	2006	8	Chilean abalone
15. Pueblo Norte Sector B	insc	89	64.15	1997	2002	13	Chilean abalone
16. Punta Cadena	insd	88	947.2	2004	2006	11	Chilean abalone
17. Los Partidos[γ]	insa	125	416	2004	2006	9	Chilean abalone
18. Puerto Sur[γ]	insb		552.79	1997	2002	13	Chilean abalone
19. Weste Isla Mocha[†]	inse	86	3870.81	1999	2003	12	Chilean abalone
20. Quechol[†]	insf		289	2003	2007	8	Chilean abalone
21. Quechol Sur	insg	31	2376.77	2003	2005	9	Chilean abalone

AFO, artisanal fisher organization.

[ψ]information until 2017.

[*]indicates the first target specie inscribed in the MEABR register, according to the management plan by SUBPESCA.

[ϕ]These management areas have the same artisanal fishers' organization: "Sindicato de Trabajadores Independientes, Pescadores Artesanales, Buzos Mariscadores y Actividades Conexas de Caleta Punta Lavapie".

[γ]These management areas have the same artisanal fishers' organization: "Cooperativa de Pescadores Pelilleros, Isla Santa Maria Limitada".

[†]These management areas have the same artisanal fishers' organization: "Organización Comunitaria Funcional de Pescadores Artesanales y Buzos Mariscadores de Isla Mocha".
