# Supplementary material for: Integrating human and ecological dimensions: The importance of stakeholders’ perceptions and participation on the performance of fisheries co-management in Chile
Source: PLoS One. 2021 Aug 11;16(8):e0254727. doi: 10.1371/journal.pone.0254727 (PMC8357100; doi:10.1371/journal.pone.0254727)
Supplement: S3 Table — (PDF) [file pone.0254727.s006.pdf]

S3 Table. Rapfish sustainability dimensions and attributes for the MEABR.

Dimension	Code	Attribute	Source
ECOLOGICAL	1. Abund_Level[g, i]	Abundance level of target specie	Secondary: SUBPESCA (technical reports), Primary: survey
	2. Other_Sp[g, i]	Re-orientation to other species	Secondary: SUBPESCA (technical reports), Primary: survey
	3. Ch_Size[g]	Changes in the mean size of target species	Secondary: SUBPESCA (technical reports)
	4. Ch_Condition[g]	Changes in the condition (wellness) index of target specie	Secondary: SUBPESCA (technical reports)
	5. Ch_Density[g]	Change of the mean density of target specie	Secondary: SUBPESCA (technical reports)
	6. Hab_Propor[g, i]	Proportion of the habitable area depending on the exploited specie	Secondary: SUBPESCA (technical reports), Primary: survey
	7. Harv_Index[g]	Harvest fulfillment index of target specie	Secondary: SUBPESCA (technical reports), Primary: survey
	8. Harv_Area[g, i]	Harvest per suitable area	Secondary: SUBPESCA (technical reports), SERNAPESCA, Primary: survey
	9. Stab_Abund[g]	Abundance stability (1/CV of abundance) of target specie	Secondary: SUBPESCA (technical reports
TECHNOLOGICAL	10.Fleet_Capacity[g, i]	Fleet capacity, variation of no. boats and active divers	Secondary: SERNAPESCA, Primary: survey
	11.Ch_Vessel_Size[g, i]	Change of fleet capacity	Secondary: SERNAPESCA, Primary: survey
	12.Ch_Practice[i]	Change of harvest practice	Primary: survey
	13.Ch_Trip_Length[i]	Change on the trip duration (hours) from fishing cove to the MEABR.	Primary: survey
	14.Ch_Cpud[g, i]	Change of catch per unit diver	Primary: survey, Secondary: SERNAPESCA
	15.Sis_Survei[i]	Type of Surveillance system applied on the MEABR	Primary: survey
	16.Other_Activ[i]	Secondary effects from other extractive activities in the MEABR's	Primary: survey
SOCIAL	17.Strength_SN[i]	Strength of social networks for actions and decisions	Primary: survey

	18.Input_Expertise[i]	Input expertise (contribution of fisher's knowledge)	Primary: survey
	19.Particip_Orga[i]	Fisher's participation in the organization	Primary: survey
	20.Rate_Member[g, i]	Quantification of the number of members, fishers	Secondary: SUBPESCA (technical reports)<br>Primary: survey
	21.Interact_Leader[i]	Level of interaction from leader	Primary: survey
	22.Replace_Leader[i]	Frequency of the leader replacement in the fisher organization	Primary: survey
	23.Educ_Level[i]	Level of education of fishers	Primary: survey
	24.Perman_MEABR[g, i]	Tenure of management area (number of years)	Primary: survey,<br>Secondary: SERNAPESCA
	25.Develop_Index[g, i]	Fishing cove development index	Secondary: SUBPESCA (technical reports),<br>Primary: survey
ETHICAL	26.Cult_Value[i]	Cultural value, relative prestige of the activity	Primary: survey
	27.Activ_Access[i]	Access to activity, facilities and historical access	Primary: survey
	28.Right_Manag[i]	Right management, participation and benefit distribution	Primary: survey
	29.Evol_Destruc[g, i]	Evolution destruction ecosystem, number of industrial establishments	Primary: survey,<br>Secondary: SUBPESCA, Superintendencia del Medio Ambiente (SMA)
	30.Vulnera_Outsi[g, i]	Vulnerability of outsiders, entry probability of outsiders	Primary: survey,<br>Secondary: SERNAPESCA
	31.Evol_Poach[i]	Evolution of illegal fishing, poaching	Primary: survey
	32.Mitigation[i]	Damage mitigation, are there any mitigation (reductions) plans?	Primary: survey
ECONOMIC	33.Econ_Production[g, i]	Economic production (quota compliance level)	Primary: survey,<br>Secondary: SERNAPESCA, SUBPESCA (technical reports)
	34.Cost_Kilo[g, i]	Cost per kilo (or unit)	Primary: survey,<br>Secondary: SERNAPESCA,<br>IFOP (technical reports)
	35.Cost_Benefit[g, i]	Cost-benefit associated to MEABR administration	Primary: survey

	36.Percap_Income[g, i]	Per capita income	Secondary: SERNAPESCA, IFOP<br>Primary: survey
	37.Alterna_Income[i]	Alternative income, alternative income	Primary: survey
	38.Subsi_Receiv[i]	Subsides received at organizational level and by member	Primary: survey
	39.Level_Of_Debt[i]	Debt level and response capacity	Primary: survey
	40.Average_Wage[i]	Average wage (or incomes) of fisher respect to minimal salary	Primary: survey
	41.Market[i]	Market, the kind of sale applied	Primary: survey
INSTITUTIONAL	42.Presen_Advice[i]	Presence and advice	Primary: survey
	43.Access_Comunic[i]	Communication access, type and frequency of communication	Primary: survey
	44.Intern_Conflict[i]	Internal conflict level, frequency and punishments applied into the organization	Primary: survey
	45.Extern_Conflict[i]	External conflict level, frequency	Primary: survey
	46.Intern_Resolve[i]	Internal conflict resolution	Primary: survey
	47.Extern_Resolve[i]	External conflict resolution	Primary: survey
	48.Network_Institu[i]	Networks with institutions	Primary: survey
	49.Network_AFO[i]	Networks with other fisher organizations	Primary: survey
	50.Goal_Fulfill[i]	Level of compliance goals	Primary: survey
	51.No_Projec[g, i]	Number of development projects	Primary: survey,<br>Secondary: SERNAPESCA

MEABR, management and exploitation area of benthic resources; AFO, artisanal fisher organization.

[g]=grupal information.

[i]=individual information.
