# Supplementary material for: Integrating human and ecological dimensions: The importance of stakeholders’ perceptions and participation on the performance of fisheries co-management in Chile
Source: PLoS One. 2021 Aug 11;16(8):e0254727. doi: 10.1371/journal.pone.0254727 (PMC8357100; doi:10.1371/journal.pone.0254727)
Supplement: S4 Table — One-way ANOVA analysis and t-test comparison between respondents (fishers) categorized. (PDF) [file pone.0254727.s007.pdf]

S4 Table. Average fishers' perception scoring for all 18 variables (see note below table for description) per each Rapfish dimension. One-way ANOVA analysis and t-test comparison between respondents (fishers) categorized

Aspects	Variable name	Category	Total fishers (n=117)	Perceptions per each dimension$^{\phi}$						Sig. diff. per each dimension (p < 0.01= '***', p < 0.05= '**', p < 0.1= '*')					
				Ecol	Tech	Soci	Ethi	Econ	Inst						
Human	1. GENDER	Female	27	4.6	7.1	6.3	5.3	5.6	6.1	***				*	***
		Male	90	5.3	7.1	6.2	5.0	5.8	5.6						
	2. CATEGORY_AGE	< 50 yrs_old	64	5.3	7.1	6.2	5.1	5.8	5.7						
		> 50 yrs_old	53	5.1	7.2	6.2	5.0	5.7	5.6						
	3. MARIT_STATUS	Married	86	5.2	7.1	6.1	5.1	5.8	5.7			**			
		Unmarried	31	5.1	7.1	6.4	5.0	5.6	5.7						
	4. LEVEL_OF_EDUC	Complete	18	5.1	7.1	6.9	5.2	5.9	5.9			***			
		Incomplete	99	5.2	7.1	6.1	5.0	5.7	5.6						
	5. AVERAGE_INCOME	< 300000 CLP	86	5.1	7.1	6.1	5.1	5.7	5.7	**				*	
		> 300000 CLP	31	5.4	7.2	6.4	5.0	5.9	5.7						
	6. CATEGORY_NDEPEND	Less than 3 persons	88	5.1	7.2	6.3	5.1	5.7	5.7			*			
		More than 3 persons	29	5.3	7.0	6.0	4.8	5.7	5.4						
	7. CATEGORY_RATIO	Low	17	4.9	7.3	6.3	5.5	5.9	5.9				**	*	
		Medium (0.5)	24	5.3	7.1	6.1	5.3	6.0	5.9						
		High	76	5.1	7.1	6.2	4.9	5.6	5.5						
Organization	8. ORGANIZATIONAL ROLE	Leader	31	5.0	7.2	6.4	5.1	5.8	5.7			**			
		Member	86	5.2	7.1	6.1	5.0	5.7	5.7						
	9. OTHER ACTIVITIES	Yes	65	5.1	7.2	6.2	5.3	5.8	5.9	*			**		**
		No	52	5.3	7.0	6.2	4.8	5.7	5.4						
	10. TYP	Union	84	4.9	7.1	6.3	5.2	5.8	5.8	***		***	***		**
		Cooperative	12	5.6	7.3	6.2	4.6	5.5	5.5						
		Other	21	5.7	7.1	5.6	4.8	5.7	5.1						
	11. CATEGORY_PERIOD	< 10 yrs.	23	4.5	7.3	6.3	5.3	5.5	6.0	***					
		11 – 19 yrs.	60	5.3	7.1	6.1	5.0	5.8	5.5						
		> 20 yrs.	34	5.3	7.1	6.2	5.0	5.8	5.7						
	12. CATEGORY_NMEABR	1 management area	57	4.8	7.1	6.3	5.1	5.6	5.6	***					
		2 management areas	38	5.5	7.1	6.1	4.9	5.8	5.7						
		3 management areas	22	5.3	7.1	6.0	5.3	6.0	5.8						
Physical	13. ACCESSIBILITY	Less than 0.5 km	65	4.9	7.2	6.3	5.2	5.7	5.7	***			**		
		More than 0.5 km	52	5.5	7.1	6.1	4.8	5.8	5.6						
	14. DEVLOP	Urban	27	5.0	6.9	6.5	4.9	5.6	5.5			**			
		Rural	90	5.2	7.2	6.1	5.1	5.8	5.7						
	15. MAIN_TARGET_SP†	Chilean abalone	103	5.3	7.1	6.1	5.0	5.8	5.5	***	*	***	***		***
		Seaweed	14	4.1	7.4	6.7	5.7	5.6	6.8						

16. ZONE	Bay	15	4.7	7.0	6.7	5.2	5.6	5.9						
	Coast	31	5.3	7.0	6.2	5.3	5.9	5.7	***		***			
	Gulf	32	4.6	7.2	6.3	5.1	5.6	5.9						
	Island	39	5.7	7.2	5.9	4.8	5.8	5.4						
17. CATEGORY_TAREA	< 250 hectares	77	5.0	7.1	6.3	5.1	5.7	5.8						
	250 – 500 hectares	17	5.1	7.2	6.1	5.2	5.6	5.4	***		**			
	> 500 hectares	23	5.7	7.2	5.8	4.7	5.9	5.4						
18. CATEGORY_EAREA	< 500 hectares	71	4.9	7.0	6.4	5.0	5.6	5.7						
	15 – 30 hectares	23	5.2	7.3	5.9	5.3	5.8	5.6	***	**	***		***	
	> 30 hectares	23	5.7	7.4	5.9	5.0	6.2	5.6						

Eco, ecological; Tech, technological; Soci, social; Ethi, ethical; Econ, economic; Inst, institutional.

ϕ Mean values. An attribute value closer to ten (10) is more favorable.

† For this analysis, despite of unbalanced sample we considered both target species, because in the first case, Chilean abalone 'loco' has a higher commercial value and in the second case, harvest records of seaweeds have increased in the last 10 years and managed mainly by women.

Notes

1. GENDER : gender of participant, grouped by female and male
2. CATEGORY_AGE : grouped of ages per fisher, < 50 years old and > 50 years old
3. MARIT_STATUS : marital status, grouped by married and unmarried
4. LEVEL_OF_EDUC : level of education, grouped by incomplete and complete
5. AVERAGE_INCOME : average income divided in two groups, < 300000 CLP (Chilean pesos) and > 300000 CLP
6. CATEGORY_NDEPEND : number dependent in household, grouped by less than 3 and more than 3 persons
7. CATEGORY_RATIO : proportion of fishing activity in general, grouped by low, medium and high (more than 0.5)
8. ORGANIZATIONAL ROLE : role of fisher inside the organization, grouped by member and leader
9. OTHER ACTIVITIES : other activities different to fishing, grouped by two options: yes or no
10. TYP : type of fisher's organization, grouped by union, cooperative and other
11. CATEGORY_PERIOD : period in years of fisher's organization with MEABR, grouped by < 10 years, 11-19 years and > 20 years
12. CATEGORY_NMEABR : number of MEABRs that fisher's organization owns, grouped by 1, 2 or 3 management areas
13. ACCESSIBILITY : distance from fishing cove base to MEABR, grouped by less than 0.5 km and more than 0.5 km
14. DEVLOP : developed of fishing cove, grouped by rural and urban
15. MAIN_TARGET_SP : main target specie in the MEABR, grouped by Chilean abalone and seaweed
16. ZONE : Zones where the MEABR is situated, grouped by bay, coast, gulf and island
17. CATEGORY_TAREA : total extension of the MEABR, grouped by <250 hectares (ha), 250-500 ha and >500 ha
18. CATEGORY_EAREA : effective area where the main specie is distributed, grouped by <15 ha, 15-30 ha and >30 ha
