# Supplementary material for: Integrating human and ecological dimensions: The importance of stakeholders’ perceptions and participation on the performance of fisheries co-management in Chile
Source: PLoS One. 2021 Aug 11;16(8):e0254727. doi: 10.1371/journal.pone.0254727 (PMC8357100; doi:10.1371/journal.pone.0254727)
Supplement: S1 Appendix — (PDF) [file pone.0254727.s008.pdf]

S1 Appendix. Questionnaire for fishers (leaders)

Part I

SECTION I: Geographical data

PROVINCE	TOWNSHIP	FISHING COVE	SECTOR

SECTION II : The Organization's data

2.1 FISHER'S ORGANIZATION NAME

2.2 ORGANIZATION CODE (RPA)

2.3 YEAR OF ESTABLISHMENT

2.4 PARTICIPATE ON THE LAST HARVEST

2.5 No. MEABRs

2.6 CONFORMATION OF THE ORGANIZATION
(specify if the MEABR had a period of inactivity)

2.7 REASONS FOR CONFORMATION OF THE ORGANIZATION

2.8 NAME OF THE MANAGEMENT AREA (MEABR)

2.9 MEABR Decree

2.10 LOCATION OF MEABR — ISLAND / COASTAL BORDER

SECTION III: Contact

3.1 NAME OF SURVEYED

3.2 TELEPHONE

3.3 E-MAIL

3.4 SEX

3.5 AGE

3.6 YEARS IN THE ORGANIZATION

3.7 ORGANIZATIONAL ROLE
PRESIDENT / VICE-PRESIDENT / SECRETARY / TREASURER / OTHERS

3.8 No. OF PERIODS IN CHARGE

3.9 THE FISHER ORGANIZATION HAS EXTERNAL STAFF (e.g., secretary, accountant, consultant, etc.) YES / NO

a. HOW MANY? b. WHAT FUNCTION DOES IT? SECRETARY / ACCOUNTANT / CONSULTANT / OTHER

3.10 No. OF PARTICIPANTS REGISTERED IN THE ORGANIZATION (indicate number) — MALES / FEMALES / TOTAL

3.11 WHAT IS THE MAIN FUNCTION OF PARTICIPANTS INTO THE ORGANIZATION? (Indicate the number to each function)

a. No. OF PARTICIPANTS BY CATEGORY
(indicate number)

ARTISANAL FISHER / SHIPOWNER / COLLECTOR
DIVER / DIVER ASSISTANT / OTHERS

b. No. OF ACTIVE PARTICIPANTS (indicate number) — MALES / FEMALES / TOTAL

c. No. OF PARTICIPANTS IN THE MEABR (indicate number) — MALES / FEMALES / TOTAL

d. No. OF ACTIVE DIVERS (indicate number)

e. No. COMPLETE ACTIVE DIVING EQUIPMENT (indicate number)

SECTION IV: Infrastructure

4.1 THE ORGANIZATION HAS AN OWN FISHING COVE 'CALETA' YES ☐ NO ☐

4.2 WHAT INFRASTRUCTURE DOES THE 'CALETA' HAVE FROM WHERE IT OPERATES IN MANAGEMENT AREA?

PIER	ESPLANADE	WINCHE	CRANE	ACCESS TO VEHICLES	TOILETS	OFFICE	STOREROOMS	STORAGE	ELECTRIC	WATER	OTHERS (SPECIFY)

4.3 ACCESSIBILITY TO FISHING COVE 'CALETA' GOOD ☐ REGULAR ☐ BAD ☐

4.4 HOW IS ACCESSIBILITY TO THE CALETA?

PAVED ROAD	ACCESS FOR TRUCKS OR HEAVY VEHICLES	CRANE, PIER OR POSSIBILITY TO ONBOARD HEAVY LOAD
LANDED RAMP		

4.5 HOW IS OVERLAND ACCESS TO THE MANAGEMENT AREA?

GOOD		REGULAR		BAD		WITHOUT ACCESS FROM LAND	

4.6 CHECK THE TYPE OF GOODS THAT THE ORGANIZATION HAS (Optional)

TYPE OF GOODS	No.	characteristic (operative or not)
boat		
engine		
diving suit		

TYPE OF GOODS	No.	characteristic (operative or not)
compressor		
others		

4.7 INDICATE SOURCE OF FUNDING ASSIGNED

GOVERNMENT ☐ % PRIVATE ☐ % CO-FINANCING ☐ % OWN ☐ %

SECTION V: Categorization

HEAD OF HOUSEHOLD

5.1 ARE YOU HEAD OF HOUSEHOLD? YES ☐ NO ☐

Detail:

a. IN WHICH FORM?
b. DO YOU ORGANIZE THE HOME?

ECONOMIC DEPENDENCE

5.2 HOW MANY PEOPLE LIVE AT HOME? ☐

5.3 HOW MANY PEOPLE DEPENDS ON YOU? ☐

MARITAL STATUS

5.4 WHAT IS YOUR MARITAL STATUS? ☐

EDUCATIONAL LEVEL

5.5 WHAT WAS YOUR LAST APPROVED COURSE? ☐

OCCUPATIONAL ACTIVITY

5.6 WHAT IS YOUR MAIN WORK WITHIN THE ARTISANAL FISHING ACTIVITY?

OCCUPATIONAL BACKGROUND

5.7 WHEN DID YOU START WITH THE ACTIVITY? (please detail some aspects) ☐

INCOME FROM LABOR ACTIVITY

5.8 IN ALL THE ACTIVITY: HOW MUCH (IN $, CHILEAN PESOS) IS YOUR AVERAGE INCOME IN THE LAST 12 MONTHS?

<$100 000
$101 000 - $250 000
$251 000 - $400 000
>$400 000

5.9 DO YOU HAVE ANOTHER ACTIVITY DIFFERENT FROM ARTISANAL FISHING? (e.g., farming, building, business, forestry, transport, etc.)

YES ☐ NO ☐

5.10 In the case of **AFFIRMATIVE** answer, mention the activity and HOW MUCH DO YOU EARN? ☐

< $100 000 $101 000 - 200 000 $201 000 - 300 000 > $ 300 000

5.11 HOW MANY PEOPLE CONTRIBUTE TO GENERATING INCOME AT HOME? ☐

4.12 WHAT IS THE TOTAL MONTHLY AVERAGE INCOME AT HOME? (includes that you perceive)

< $200 000
$201 000 - $300 000
$301 000 - $400 000
> $400 000

SECTION VI: Organizational planning and training

6.1 DOES THE ORGANIZATION HAVE **ANNUAL OBJECTIVES**? YES ☐ NO ☐

6.2 CURRENTLY AND ACCORDING TO YOUR CRITERIA, WHAT IS **THE LEVEL OF FULFILLMENT** ABOUT SUCH **OBJECTIVES**?

MORE THAN 60% ☐ BETWEEN 40% AND 60% ☐ LESS THAN 30% ☐

6.3 HAVE MEMBERS BEEN TRAINED DURING THE LAST 5 YEARS?

YES ☐ NO ☐ Indicate, what are the reasons? ☐

if it is **AFFIRMATIVE**, it could detail the following:

a. INDICATE TYPE OF TRAINING

COMMERCIALIZATION ☐ SAMPLING ☐ AQUACULTURE ☐ ENVIRONMENT ☐ OTHER ☐

b. HOW MANY MEMBERS PARTICIPATED IN THE TRAINING?

LESS THAN 30% ☐ BETWEEN 30% AND 50% ☐ MORE THAN 60% ☐

c. HAVE THE TRAININGS IMPROVED THE PERFORMANCE OF MEMBERS IN THE MANAGEMENT AREA?

YES ☐ NO ☐ WHY? ☐

SECTION VII: Participation

7.1 HOW OFTEN ARE THE MEETINGS WITH MEMBERS OF THE FISHER ORGANIZATION?

MONTHLY ☐ EACH 2 MONTHS ☐ MORE THAN 2 MONTHS ☐

7.2 WHEN THERE IS AN **IMPORTANT OR URGENT INFORMATION** TO BE DELIVERED AT MEMBERS, HOW IS **TRANSMITTED IT**?

EXTRAORDINARY MEETING ☐ INFORMALLY ☐ PUBLISHED OR SPREAD ☐ OTHER ☐

7.5 DO YOU PARTICIPATE IN FEDERATION MEETINGS OR FISHER ASSOCIATIONS AT LOCAL LEVEL?

YES ☐ NO ☐ WHICH? ☐

7.6 WHAT IS YOUR **MAIN ROLE** IN THESE MEETINGS? (AFO = Artisanal fisher organization, AF = Artisanal fisher)

AFO REPRESENTATIVE ☐ FEDERATION OR ASSOCIATION ☐ TYPE OF CHARGE ☐

7.7 HOW OFTEN IS THERE CHANGE OF LEADERSHIP?

EACH 2 YEARS ☐ EACH 4 YEARS ☐ MORE THAN 4 YEARS ☐

SECTION VIII: Relationship with the surroundings

8.1 DOES THE ORGANIZATION PARTICIPATE IN MIXED COMMISSIONS (i.e., PUBLIC-PRIVATE) LINKED TO FISHERIES SECTOR?

YES ☐ NO ☐ if the answer is **AFFIRMATIVE**, detail in which commissions? ☐

8.2 HAS THE ORGANIZATION BEEN BENEFITED BY PARTICIPATING IN THESE COMMISSIONS (e.g., projects, financing, subsides, etc.)?

YES ☐ NO ☐ if the answer is **AFFIRMATIVE**, indicate the type of benefits ☐

8.3 DOES THE ORGANIZATION KEEP NETWORKS OF COOPERATION WITH OTHER FISHER ORGANIZATIONS?

YES ☐ NO ☐ if the answer is **AFFIRMATIVE**, indicate the number of cooperation networks, with whom? ☐

8.4 ACCORDING TO YOUR OPINION, KEEPING NETWORKS WITH OTHER AFOs* HAS LEADED BENEFITS TO YOUR ORGANIZATION?

YES ☐ NO ☐ if the answer is **AFFIRMATIVE**, indicate benefits ☐

*AFO = Artisanal fisher organization

SECTION IX: Activity in the MEABR

9.1 HOW MANY BOATS AND DAYS OPERATE DURING THE **"HARVEST SEASON"**? ☐ ☐ days

9.2 HOW ARE THE INCOME GENERATED BY **FISHING HARVEST** AMONG PARTICIPANTS DISTRIBUTED?

☐

9.3 IN CASE REPORTING INACTIVE MEMBERS, HOW MUCH INCOME DOES IT PERCEIVE RESPECT TO AN ACTIVE MEMBER?

NOTHING ☐ LESS THAN 30% ☐ BETWEEN 30% AND 50% ☐ MORE THAN 60% ☐ 100% ☐

SECTION X: Operating costs

10.1 INDICATE COSTS ($, CHILEAN PESOS) ASSOCIATED TO OPERATION INTO THE MEABR

item	annual value ($ / year)
surveillance	
monitoring study	
marketing (transportation of goods)	

item	annual value ($ / year)
rent	
others* (specify)	

(*) remodeling costs, fishing pier, vehicles, etc.

10.2 INDICATE COSTS ASSOCIATED WITH FUNCTIONING OF THE ARTISANAL FISHER ORGANIZATION

item	annual value ($ / year)
secretary	
basic services	
marketing (goods, others)	

item	annual value ($ / year)
rent	
maintenance	
others (specify)	

10.3 EXPENSES ASSOCIATED WITH THE "HARVEST SEASON"

item	liters	annual value ($ / year)
fuel		
oil		
others (specify)		

10.4 DOES THE ORGANIZATION RECEIVE OTHER INCOME? YES ☐ NO ☐

if the answer is **AFFIRMATIVE**, indicate the value of the income per item

item	annual value ($ / year)	observations
service of fishing pier and infrastructure		
sport fishing		
rents (restaurants, kiosks, trade fairs, fishmongers, etc.)		
parking lots		
turistic services (boat sailing, diving into MEABRs, etc.)		
other incomes		

10.5 DURING THE LAST YEAR, HAS THE FISHER ORGANIZATION GIVEN MONEY TO MEMBERS? HOW MUCH MONEY IN AVERAGE PER MEMBER?

YES ☐ NO ☐ if the answer is **AFFIRMATIVE**, indicate how much to each member (an estimate) ☐

SECTION XI: Commercialization

11.1 DOES THE ORGANIZATION CARRY OUT ANY TYPE OF PROCESS TO RESOURCES BY GENERATE A BEST SALE PRICE?

YES ☐ NO ☐ if the answer is **AFFIRMATIVE**, indicate the type of process ☐

11.2 HOW IS THE NEGOTIATION SYSTEM OF THE "HARVEST"?

a.- consultation from leaders to members	
c.- a commission by agreement with members	
e.- it is not negotiated, it is fixed by the buyer (intermediary)	

b.- only leaders without consulting	
d.- a commission, without consulting	
f.- others	

11.3 WHAT IS THE MARKET? DIRECT SALE ☐ SALE TO PROCESSING PLANT ☐ INTERMEDIARY ☐

Comments:

11.4 DOES THE ORGANIZATION SELL IN CONJUNCTION WITH OTHER FISHERS' ORGANIZATIONS?

YES ☐ NO ☐ if the answer is **AFFIRMATIVE**, indicate the number of fisher organizations ☐

11.5 WHAT ARE SALE PRICES (IN AVERAGE) PER **"HARVESTED"** RESOURCES IN THE MEABR? Indicate the value ($) per kilo or unit (list in order of importance by commercial species in the AMERB)

species	jan-mar (kilo or unit)	apr-jun (kilo or unit)	jul-sep (kilo or unit)	oct-dec (kilo or unit)

SECTION XII: Project Advice and Development

12.1 INDICATE, WHICH ENTITIES **ACTIVELY PARTICIPATE OR SUPPORT** IN THE **SURVEILLANCE** OF YOUR MEABR?

SERNAPESCA ☐ COAST GUARD ☐ PRESENCE OF SOME AUTHORITY (POLICE) ☐ NOTHING ☐

OTHER: ☐

12.2 HAS YOUR ORGANIZATION CARRIED OUT ASSOCIATED PROJECTS TO DEVELOPMENT OF MANAGEMENT AREA DURING THE LAST YEAR?

YES ☐ NO ☐ if the answer is **AFFIRMATIVE**, list implemented projects ☐

12.3 WHO WAS THE MAIN CONTRIBUTOR OF FINANCING FOR IMPLEMENTED PROJECTS?

GOVERNMENT ☐ % PRIVATES ☐ % CO-FINANCING ☐ % OWNS ☐ %

12.4 IS THERE CO-FINANCIAL SUPPORT FOR PROJECTS MANAGED BY YOUR FISHER ORGANIZATION?

YES ☐ NO ☐

IF AFFIRMATIVE, HOW MANY **PROJECTS HAVE BEEN EXECUTED** IN THE LAST 5 YEARS?

MORE THAN ONE PROJECT ☐ EQUALS A ONE PROJECT ☐ NONE ☐

GLOBAL PERCEPTION FROM MANAGEMENT AREA - MEABR (open question)

13.1 GIVEN THE **PERFORMANCE OF MEABR** THAT HAVE YOUR ORGANIZATION, **WHY DO YOU AGREE TO KEEP IT?**

13.2 ACCORDING TO YOUR PERCEPTION, WHAT ARE THE **MAIN ADVANTAGES** (strengths, benefits) AND **DISADVANTAGES** (weaknesses, problems) OF THE MEABR ASSIGNED TO YOUR ORGANIZATION?

13.3 ON THE PERSONAL, WHAT DO **YOU EXPECT** FROM THE MEABRs **IN NEXT YEARS**?

Part II

SECTION I: Ecological-fishery indicator

ABUNDANCE LEVEL AND VARIATION OF TARGET SPECIES

1.1 WHAT ARE THE COMMERCIAL SPECIES HARVESTED IN YOUR MANAGEMENT AREA? YOU COULD DETAIL AT WHAT LEVEL IS EACH ONE IN THE LAST 5 YEARS: PRODUCTIVE (+), MAINTAINED (=) OR DEPRESSED (-). Please, order from the most important to least amount

SPECIE	+	=	-	%
A.				
B.				
C.				
D.				
E.				

SPECIE	+	=	-	%
F.				
G.				
H.				
I.				
J.				

1.2 IN YOUR MEABR, THERE HAVE BEEN REORIENTED TO OTHER SPECIES IN THE LAST 5 YEARS, WHAT ARE THOSE SPECIES?

Before proceeding to the **next question (1.3)**, consult if they have notions on the subject. Otherwise, it is aimed at divers of the fisher organization.

FRACTION OF HABITABLE AREA

1.3 IN THE LAST 5 OR 10 YEARS INDICATE IN PERCENTAGE, HOW HAS THE HABITABLE SUBSTRATE CHANGED TO THE MAIN SPECIES?
(if necessary, show a map of the management area)

Propor. (%)			%		%		%
	HARD SUBSTRATE AREA :	INCREASE		CONSTANT		DECREASE	
	SEMI-HARD SUBSTRATE AREA :	INCREASE		CONSTANT		DECREASE	
	SOFT SUBSTRATE AREA :	INCREASE		CONSTANT		DECREASE	

Detail:

SECTION II: Technological Indicator

FLEET CAPACITY (No. of boats or active divers)

2.1 HOW THE NUMBER OF BOATS AND ACTIVE DIVERS HAS MAINTAINED IN THE MEABR BEFORE AND AFTER 2010*?, AND INDICATE THE No. APPROX. OF BOATS AND DIVERS ACCORDING TO THEIR ORIGIN, WHICH CARRY OUT FISHING ACTIVITIES. (*EARTHQUAKE AND TSUNAMI 27F)

DETAIL No OF BOATS AND DIVERS		before 2010	after 2010		before 2010	after 2010		
no. boats	local			outside			origin	
no. divers (active condition)	local			outside			origin	

CHANGE OF VESSEL SIZE

2.2 WHAT IS THE **CAPACITY IN METERS (LENGTH)** OR TONNAGE OF BOAT THAT WORKS IN THE MEABR? Detail some features: material, type of engine, among others

a. BOAT CHARACTERISTICS

years		max. length		tonelage (GRT)	
material		max. width		others	
type of engine					

b. HAS THE SIZE OF BOATS (IN METERS OR GRT) VARIED IN THE LAST 10 YEARS?

INCREASED ☐ KEEP CONSTANT ☐ DECREASED ☐

If there is VARIATION (INCREASE OR DECREASE), IN WHICH PERCENTAGE?

< 5% ☐ between 5% and 19% ☐ between 20% and 49% ☐ > 50% ☐

RECENT CHANGES OF HARVEST PRACTICE

2.3 IN THE LAST 5 YEARS, HAS CHANGES BEEN OBSERVED ON USE OF **NEW FISHING GEARS** IN THE MEABRs?

a. CHANGES IN THE USE OF FISHING GEARS BY HARVEST IN THE MEABR

NONE, LITTLE CHANGE (< 5%) ☐ GRADUAL CHANGE (between 5% and 49%) ☐ PARTIAL CHANGE (between 50% and 99%) ☐ FULL (> 100%) ☐

b. WITH WHICH IMPLEMENTS DOES IT BE ACCOUNT? (If there are others, detail):

boat without accessories ☐ boat with accessories ☐

fishing implement	quantity	unit price $	age
out. propulsion engine			
compressor			
regulator			
diving belt			

fishing implement	quantity	unit price $	age
life vest			
dive viewer			
diving suit rubber			
diving hose			

comments:

CHANGE OF TRIP DURATION

2.4 HOW LONG TIME (TRIP HOURS) THE BOAT SPEND FROM FISHING COVES TO MEABR? OR THE DISPLACEMENT DISTANCE (KM)?

a. IN HOURS:

LESS THAN 1 hr ☐ 2-4 hrs ☐ 5-8 hrs ☐ MORE THAN 8 hrs ☐

b. IN KILOMETERS

LESS THAN 1 km ☐ 1-5 km ☐ 5-10 km ☐ MORE THAN 10 km ☐

SURVEILLANCE SYSTEM

2.5 HOW THE SURVEILLANCE SYSTEM HAS BEEN CARRIED OUT BY YOUR ORGANIZATION IN THE MEABR?

FULL IMPLEMENTED ☐ (camera and others) BASIC SYSTEM ☐ (radio, binocular, others) AUTO-SURVEILLANCE ☐ NON-EXISTENT ☐

comments:

OTHER ACTIVITIES

2.6 HOW OFTEN IS THE INCIDENCE OF OTHER ACTIVITIES **THAT HAVE SIDE EFFECTS IN THE MEABR**?. For example, fishing activities, drainage, aquaculture, and others.

A LOT ☐ SOME ☐ NONE ☐

SECTION III: Social indicator

STRENGTH OF SOCIAL NETWORKS

3.1 HOW THE INFORMATION EXCHANGE HAS BEEN CARRIED OUT FOR THE DECISION-MAKING AMONG MEMBERS INTO THE ORGANIZATION?

STRONG CAPACITY INFORMATION / AGREEMENTS AMONG MEMBERS ☐ AGREEMENTS BUT REGULAR MECHANISMS ☐ DEFICIENT CAPACITY /MINORITY AGREEMENTS ☐

CONTRIBUTION OF FISHER´S KNOWLEDGE

3.2 HOW IS YOUR GENERAL KNOWLEDGE ABOUT FISHING RESOURCES AND ITS ENVIRONMENT LINKED TO MANAGEMENT AREAS?

CONSIDERABLE ☐ ENOUGH ☐ SOMETHING SUFFICIENT ☐ LITTLE ☐ VERY LITTLE ☐

CHANGE RATE IN THE NUMBER OF MEMBERS

3.3 IN THE LAST 10 YEARS, HOW THE MEMBERS NUMBER HAVE VARIED IN THE ORGANIZATION?
(relate the number of currently participants, compared to previous years)

INCREASED (proportion > 3) ☐ SAME (proportion = 1) ☐ DECREASED (propotion < 1) ☐

GENDER ROLE (*)

3.4 IN THE LAST YEARS, HOW OFTEN DO **WOMEN PARTICIPATE AS MEMBER** IN THE ORGANIZATION?

VERY FREQUENT ☐ FREQUENT ☐ OCCASIONALLY ☐

RARELY ☐ NEVER ☐

3.5 BY OTHER SIDE, HOW DO YOU CLASSIFY **WOMEN'S PERFORMANCE** IN THE ORGANIZATION?

VERY GOOD ☐ GOOD ☐ REGULAR ☐ BAD ☐ VERY BAD ☐

details:

SECTION IV: Ethic indicator

CULTURAL VALUE

4.1 IN YOUR OPINION, WHAT DO YOU EXPECT FROM THIS OFFICE LINKED TO OWN ACTIVITY FROM MEABR FOR THE FUTURE GENERATIONS?

CULTURAL IDENTITY AND DIVERSIFY ☐ ONLY AS AN **ALTERNATIVE ACTIVITY** ☐ YOUNG PEOPLE **DO NOT FIND IT AS AN ATTRACTIVE** JOB ☐

RIGHT MANAGEMENT

4.2 ACCORDING YOUR PERCEPTION, DECISION-MAKING IN ALL THE SYSTEM. HOW IT HAS BEEN CARRIED OUT IN THE LAST YEARS?
(e.g., how make-decision about the fishing? and so on)

EQUALLY PARTICIPATION INCLUDING FISHERS ☐ GOVERNMENT WITH SCIENTIFIC ADVISING ☐ ONLY GOVERNMENT ☐

EVOLUTION DESTRUCTION ECOSYSTEM

4.3 HOW THE NUMBER OF INDUSTRIAL ESTABLISHMENTS HAS EVOLVED NEAR TO MEABR?

(+)
NO INCREASED / WASTE CONTROL ☐ INCREASE / WASTE CONTROL ☐ NO INCREASED / POOR CONTROL ☐

INCREASED / ZERO CONTROL ☐ POLLUTION ☐
(-)

VULNERABILITY TO OUTSIDERS

4.4 HOW FREQUENT IS THE ENTRANCE OF OUTSIDER BOATS (INCLUDE DIVERS) TO MEABR?

NULL ☐ REGULAR ☐ FREQUENTLY ☐
(VULNERABLE TO OUTSIDERS' ENTRANCE)

EVOLUTION OF ILLEGAL FISHING (POACHING)

4.5 HOW DO YOU CATEGORIZE **ILLEGAL FISHING** IN THE MEABR?. This includes several illicit activities: Fishing without permission, don´t respect to catch quotas, don´t declare or give false information about harvest among others.

NONE ☐ SOME, HAVE RULES TO REDUCE POACHING ☐ TOO MUCH (ILLEGAL FISHING) ☐

DAMAGE MITIGATION PROGRAM

4.6 IS THERE ANY MANAGEMENT PLAN TO MINIMIZE NEGATIVES ENVIROMENTAL IMPACTS THAT OCCUR INTO THE MEABR?

YES ☐ NO ☐

4.7 If it is **AFFIRMATIVE**, HOW WAS IT CARRIED OUT?

EFFECTIVE / IMPLEMENTATION PROGRAM ☐ SOME OF MITIGATION MEASUREMENT ☐ NONE ☐

SECTION V: Economic indicator

COST-BENEFIT

5.1 ACCORDING TO COSTS (OPERATIVE AND VARIABLE) THAT GENERATE IN THE MEABR, HOW DO YOU CLASSIFY (PROPORTION= Benefit/Cost)?

MEABR GENERATES PROFIT (proportion B/C > 1) ☐ IT HAS REMAINED STABLE ☐ MEABR GENERATES LOSSES (proportion B/C < 1) ☐

INDIRECT TOTAL INCOME

5.4 HOW DO YOU CLASSIFY THE **FISHING ACTIVITY IN THE MEABR** RESPECT TO OTHER ACTIVITIES CARRIED OUT BY THE ORGANIZATION?

MAIN ACTIVITY ☐ COMPLEMENTARY ACTIVITY ☐ MARGINAL ACTIVITY ☐

comments:

DEBT LEVEL

5.3 DO YOU HAVE KNOW ABOUT S**OME DEBT THAT YOUR ORGANIZATION** HAS WITH FINANCIAL ENTITIES? YES ☐ NO ☐

If the answer is **AFFIRMATIVE**, continue with 5.5

approximate average amount of debt (optional) $

5.4 IN THE LAST 5 YEARS, HOW DO YOU CATEGORIZE THE DEBT LEVEL (=Annual debt/Total income)?

OPTIMAL DEBT LEVEL (<40%) ☐ RISKY DEBT LEVEL (> 40%) ☐

SECTION VI: Institucional indicator

INTERNAL CONFLICTS LEVEL

6.1 HOW DO YOU QUALIFY THE AMOUNT OF CONFLICTS GENERATED INTO YOUR ORGANIZATION?

VERY FREQUENT (> 60%) ☐ REGULAR (BETWEEN 40% AND 60%) ☐ NULL (<= 30%) ☐

6.2 INDICATE THE FOLLOWING:

No. of sanctions

In how long time?

detail, WHAT KIND OF **SANCTIONS** HAVE BEEN APPLIED?

EXTERNAL CONFLICTS LEVEL

6.3 HOW DOES THE AMOUNT OF CONFLICTS GENERATE OUTSIDE YOUR ORGANIZATION? (e.g., neighbors of the organization, public, and private actors)

HIGH (> 3 ORGANIZATIONS OR INSTITUTIONS) ☐ MEDIUM (BETWEEN 1 AND 2) ☐ LOW (< 1) ☐

6.4 LIST, WHAT KIND OF CONFLICTS HAVE BEEN GENERATED?

CONFLICT RESOLVE MECHANISM

6.5 ARE THERE ANY MECHANISMS TO RESOLVE CONFLICTS INTO YOUR ORGANIZATION?

YES, VERY EFECTIVE ☐ EXISTS, BUT IT ISN'T EFFECTIVE ☐ NULL ☐

6.6 DETAIL, WHAT ARE THESE MECHANISMS? IF NOT EXIST, WHY REASONS?

6.7 ARE THERE ANY MECHANISMS TO RESOLVE CONFLICTS OUTSIDE YOUR ORGANIZATION?

YES, VERY EFECTIVE ☐ EXISTS, BUT IT ISN'T EFFECTIVE ☐ NULL ☐

6.8 DETAIL, WHAT ARE THESE MECHANISMS? IF NOT EXIST, WHY REASONS?
