# Supplementary material for: Integrating human and ecological dimensions: The importance of stakeholders’ perceptions and participation on the performance of fisheries co-management in Chile
Source: PLoS One. 2021 Aug 11;16(8):e0254727. doi: 10.1371/journal.pone.0254727 (PMC8357100; doi:10.1371/journal.pone.0254727)
Supplement: S2 Appendix — (PDF) [file pone.0254727.s009.pdf]

S2 Appendix. Questionnaire for fishers (members)

Part I

SECTION I: Geographical data

PROVINCE	TOWNSHIP	FISHING COVE	SECTOR

SECTION II : The Organization's data

2.1 FISHER ORGANIZATION NAME

2.2 ORGANIZATION CODE (RPA)

2.3 YEAR OF ESTABLISHMENT **2.4 PARTICIPATE ON THE LAST HARVEST** **2.5 No. MEABRs**

(specify if the management area had period of inactivity)

SECTION III : Contact

3.1 NAME OF SURVEYED

3.2 SEX **3.3 AGE** **3.4 YEARS IN THE ORGANIZATION**

3.5 TELEPHONE **3.6 E-MAIL**

SECTION IV : Categorization

HEAD OF HOUSEHOLD

4.1 ARE YOU HEAD OF HOUSEHOLD? YES NO

Detail:

a. IN WHICH FORM?
b. DO YOU ORGANIZE THE HOME?

ECONOMIC DEPENDENCE

4.2 HOW MANY PEOPLE LIVE AT HOME?

4.3 HOW MANY PEOPLE DEPENDS ON YOU?

MARITAL STATUS

4.4 WHAT IS YOUR MARITAL STATUS?

EDUCATIONAL LEVEL

4.5 WHAT WAS YOUR LAST APPROVED COURSE?

OCCUPATIONAL ACTIVITY

4.6 WHAT IS YOUR MAIN WORK WITHIN THE ARTISANAL FISHING ACTIVITY?

OCCUPATIONAL BACKGROUND

4.7 WHEN DID YOU START WITH THE ACTIVITY? (please detail some aspects)

INCOME FROM LABOR ACTIVITY

4.8 IN ALL THE ARTISANAL FISHING ACTIVITY: HOW MUCH (IN $, CHILEAN PESOS) IS YOUR AVERAGE INCOME IN THE LAST 12 MONTHS?

<$100 000
$101 000 - $250 000
$251 000 - $400 000
>$400 000

4.9 DO YOU HAVE ANOTHER ACTIVITY DIFFERENT FROM ARTISANAL FISHING? (e.g., farming, building, business, forestry, transport, and so on)

YES ☐ NO ☐

4.10 In the case of **AFFIRMATIVE** answer, mention the activity and HOW MUCH DO YOU EARN? ☐

< $100 000 $101 000 - 200 000 $201 000 - 300 000 > $ 300 000

4.11 HOW MANY PEOPLE CONTRIBUTE TO GENERATING INCOME AT HOME? ☐

4.12 WHAT IS THE TOTAL MONTHLY AVERAGE INCOME AT HOME? (includes that you perceive) < $200 000 $301 000 - $400 000 $201 000 - $300 000 > $400 000

SECTION V : Productive activity

5.1 HOW MANY PEOPLE WORK BY BOAT? ☐

5.2 WHAT ARE THE MAIN SPECIES AND VOLUMES LANDED?
(could detail the price 'precio playa' and annual landings)

SPECIE	UNIT /KILO / BOX	PRICE ($)	ANNUAL LANDING*
1.-			
2.-			
3.-			
4.-			
5.-			
6.-			
7.-			
8.-			
9.-			
10.-			

*If it is neccessary

SECTION VI: Activity in the MEABR

6.1 ACCORDING TO YOUR CRITERIA, HOW THE PRODUCTIVE ACTIVITY PRE AND POST-MEABR HAS CHANGED? (it means, from MEABR was assigned at your organization)

6.2 HOW MANY BOATS AND DAYS ARE OPERATE DURING **THE "HARVEST SEASON"**? ☐ ☐ days

6.2 DURING THE **"HARVEST SEASON"**, HOW MUCH ($, CHILEAN PESOS) IS THE COST IN AVERAGE? (list the items mentioned and if there are other, add them)

items	liters/units	amount ($) / departure	annual operating costs / boat ($)
outboard motor mix			
compressor fuel			
boat supplies			

items	liters/units	amount ($) / departure	annual operating costs / boat ($)
talc diving suit			
others			
no. operative boats			

6.3 ABOUT YOUR ACTIVITY IN THE MANAGEMENT AREA, HOW MUCH IS YOUR AVERAGE INCOME?. Please, indicate an approximate value

income level	monthly (12 months)	"harvest season"
less than $50,000		
$ 51,000 - $ 100,000		
$101,000 - $150,000		
$151,000 - $200,000		
$201,000 - $250,000		
more than $251,000		

6.4 DURING THE **"HARVEST SEASON"**, HOW IS THE INCOME DISTRIBUTION SYSTEM BY PARTICIPANT?

	✓		participation	
task	respondent	total* (No.)	parts system	% parts system
crew member				
diver				
shipowner				
diver assistant				
others				

(*) participants

details:

Third party contribution

6.5 DURING THE LAST YEAR, HAVE YOU RECEIVED ANY SUBSIDY FROM GOVERNMENT, PRIVATE OR OTHER? YES ☐ NO ☐

In the case of **AFFIRMATIVE** answer, WHAT SUBSIDIES HAS BEEN RECEIVED? (agregate the approximate amount)

PER MEMBER	GROUP (IN THE FISHER ORGANIZATION)
services (for example, basic services, bonus, etc.)	productive (for example, infrastructure, equipment, materials, etc.)

Commercialization

6.6 HOW IS THE **NEGOTIATION SYSTEM** OF THE "HARVEST"?

a.- consultation from leaders to members		b.- only leaders without consulting	
c.- a commission by agreement with members		d.- a commission, without consulting	
e.- it is not negotiated, it is fixed by buyer (intermediary)		f.- others	

6.7 WHAT IS THE MARKET? DIRECT SALE ☐ SALE TO PROCESSING PLANT ☐ INTERMEDIARY ☐

comments:

Participation

6.8 DO YOU **ACTIVELY PARTICIPATE** IN THE ORGANIZATION? (detail, HOW DOES IT CONTRIBUTE?)

If **NEGATIVE** your answer, indicate the reasons:

6.9 HOW OFTEN DO YOU ATTEND THE MEETINGS CALLED BY THE ORGANIZATION?

FREQUENTLY ☐ SPORADIC ☐ LIMITED ☐

6.10 REGARDING DECISION-MAKING, PARTICIPATION, AGREEMENTS IN THE ORGANIZATION, HOW IS IT EFFECTED?
(+)
WORK TOGETHER / AND **SHARE DECISIONS** ☐

THE LEADERSHIP **ALLOWS MEMBERS** TO **MAKE DECISIONS** ☐

THERE IS **INTERACTION /** BUT **LEADERSHIP MAKES** DECISION ☐

ONLY **LEADERS TAKE DECISIONS** / **MINIMAL INTERACTION** ☐

THE LEADERSHIP HAS **MOST CONTROL** (-) ☐

6.11 WHAT IS YOUR ACTIVITY IN THE ORGANIZATION?

shipowner		diver assistants	
flaker (desconchador)		administration	
skipper		ground support work	
fisherman		shore collector	
diver			
details:			

SECTION VII: Management quality of the leaders

7.1 DO YOU KNOW PERSONALLY ABOUT THE CURRENT LEADERS OF THE ORGANIZATION?

ALL ☐ SOME ☐ NONE ☐

7.2 FROM THE PREVIOUS QUESTION, DO YOU KNOW THE WORK DEVELOPED BY THE LEADERSHIP?

YES, VERY GOOD ☐ VERY LITTLE ☐ REALLY UNKNOWN ☐

7.3 AND, HOW DOES THE LABOR PERFORMED BY CURRENT LEADERS OF THE ORGANIZATION QUALIFY?

VERY BAD ☐ BAD ☐ REGULAR ☐ GOOD ☐ VERY GOOD ☐ DO NOT KNOW / NOT ANSWER ☐

7.4 ON THE OTHER HAND, THE REPLACEMENT OF LEADERS IN THE ORGANIZATION IS:

EVERY 2 YEARS (1 PERIOD) ☐ EVERY 4 YEARS (2 PERIODS) ☐ MORE THAN 4 YEARS (> 3 PERIODS) ☐

SECTION VIII: Associativity and relationship of the organization with its surroundings

8.1 HOW DO YOU QUALIFY THE LINKAGES OF YOUR ORGANIZATION WITH OTHER ORGANIZATIONS, PRIVATE SECTOR AND GOVERNMENT?

ERY GOOD ☐ GOOD ☐ REGULAR ☐ BAD ☐ VERY BAD ☐ DO NOT KNOW / NOT ANSWER ☐

Why?

8.2 IN RELATION TO COMMUNICATION LEVEL OF YOUR ORGANIZATION WITH ITS SURROUNDINGS, IN WHAT LEVEL ARE?

type	increase	maintain	decrease	do not know/not answer
level of communication with SERNAPESCA				
level of communication with SUBPESCA				
level of communication with CONSULTANTS				
level of communication between the organization's members				
level of communication with other AFOs*				

*AFO= Artisanal fisher organization

GLOBAL PERCEPTION FROM MANAGEMENT AREA - MEABR (open question)

9.1 IN GENERAL, HOW DO YOU **DESCRIBE THE OPERATION OF YOUR MEABR?**

9.2 ACCORDING YOUR PERCEPTION, WHAT ARE THE **MAIN ADVANTAGES** (strengths, benefits) AND **DISADVANTAGES** (weaknesses, problems) OF THE **MEABR** ASSIGNED TO YOUR ORGANIZATION?

9.3 ON THE PERSONAL, WHAT DO YOU **EXPECT** FROM THE **MEABR IN NEXT YEARS?**

Part II

SECTION I: Ecological-fishery indicator

ABUNDANCE LEVEL AND VARIATION OF TARGET SPECIES

1.1 WHAT ARE THE COMMERCIAL SPECIES HARVESTED IN YOUR MANAGEMENT AREA? YOU COULD DETAIL, AT WHAT LEVEL IS EACH ONE IN THE LAST 5 YEARS: PRODUCTIVE (+), MAINTAINED (=) OR DEPRESSED (-). Please, order from the most important to least amount

SPECIE	+	=	-	%
A.				
B.				
C.				
D.				
E.				

SPECIE	+	=	-	%
F.				
G.				
H.				
I.				
J.				

1.2 IN YOUR MEABR, THERE HAVE BEEN REORIENTED TO OTHER SPECIES IN THE LAST 5 YEARS, WHAT ARE THOSE SPECIES?

Before proceeding to the **next question (1.3)**, consult if they have notions on the subject. Otherwise, it is aimed at divers of the fisher organization.

FRACTION OF HABITABLE AREA

1.3 IN THE LAST 5 OR 10 YEARS INDICATE IN PERCENTAGE, HOW HAS THE HABITABLE SUBSTRATE CHANGED TO MAIN SPECIES?
(if necessary, show a map of the management area)

Propor. (%)			%				%
	HARD SUBSTRATE AREA :	INCREASE		CONSTANT		DECREASE	
	SEMI-HARD SUBSTRATE AREA :	INCREASE		CONSTANT		DECREASE	
	SOFT SUBSTRATE AREA :	INCREASE		CONSTANT		DECREASE	

Detail:

SECTION II: Technological Indicator

FLEET CAPACITY (No. of boats or active divers)

2.1 HOW THE NUMBER OF BOATS AND ACTIVE DIVERS HAS MAINTAINED IN THE MEABR BEFORE AND AFTER 2010*?, AND INDICATE THE No. APPROX. OF BOATS AND DIVERS ACCORDING TO THEIR ORIGIN, WHICH CARRY OUT FISHING ACTIVITIES. (*EARTHQUAKE AND TSUNAMI 27F)

DETAIL No. OF BOATS AND DIVERS		before 2010	after 2010		before 2010	after 2010		
no. boats	local			outside			origin	
no. divers (active condition)	local			outside			origin	

CHANGE OF VESSEL SIZE

2.2 WHAT IS THE CAPACITY IN METERS (LENGTH) OR TONNAGE OF BOAT THAT WORKS IN THE MEABR? Detail some features: material, type of engine, among others

a. BOAT CHARACTERISTICS

years		max. length		tonelage (GRT)	
material		max. width		others	
type of engine					

b. HAS THE SIZE OF BOATS (IN METERS OR GRT) CHANGED IN THE LAST 10 YEARS?

INCREASED ☐ KEEP CONSTANT ☐ DECREASED ☐

If there is VARIATION (INCREASE OR DECREASE), IN WHICH PERCENTAGE?

< 5% ☐ between 5% and 19% ☐ between 20% and 49% ☐ > 50% ☐

RECENT CHANGES OF HARVEST PRACTICE

2.3 IN THE LAST 5 YEARS, HAS CHANGES BEEN OBSERVED ON USE OF **NEW FISHING GEARS** IN THE MEABR?

a. CHANGES IN THE USE OF FISHING GEARS BY HARVEST IN THE MEABR

NONE, LITTLE CHANGE (< 5%) ☐ GRADUAL CHANGE (between 5% and 49%) ☐ PARTIAL CHANGE (between 50% and 99%) ☐ FULL (> 100%) ☐

b. WITH WHICH IMPLEMENTS DOES IT BE ACCOUNT? (If there are others, detail):

boat without accessories ☐ boat with accessories ☐

fishing implement	quantity	unit price $	age
out. propulsion engine			
compressor			
regulator			
diving belt			

fishing implement	quantity	unit price $	age
life vest			
dive viewer			
diving suit rubber			
diving hose			

comments:

CHANGE OF TRIP DURATION

2.4 HOW LONG TIME (TRIP HOURS) THE BOAT SPEND FROM FISHING COVES TO MEABR? OR THE DISPLACEMENT DISTANCE (KM)?

a. IN HOURS:

LESS THAN 1 hr ☐ 2-4 hrs ☐ 5-8 hrs ☐ MORE THAN 8 hrs ☐

b. IN KILOMETERS

LESS THAN 1 km ☐ 1-5 km ☐ 5-10 km ☐ MORE THAN 10 km ☐

SURVEILLANCE SYSTEM

2.5 HOW THE SURVEILLANCE SYSTEM HAS BEEN CARRIED OUT BY YOUR ORGANIZATION IN THE MEABR?

FULL IMPLEMENTED ☐ (camera and others)   BASIC SYSTEM ☐ (radio, binocular, others)   AUTO-SURVEILLANCE ☐   NON-EXISTENT ☐

comments:

OTHER ACTIVITIES

2.6 HOW OFTEN IS THE INCIDENCE OF OTHER ACTIVITIES **THAT HAVE SIDE EFFECTS IN THE MEABR**?. For example, fishing activities, drainage, aquaculture, and others.

A LOT ☐   SOME ☐   NONE ☐

SECTION III: Social indicator

STRENGTH SOCIAL NETWORKS

3.1 HOW THE INFORMATION EXCHANGE HAS BEEN CARRIED OUT FOR THE DECISION-MAKING AMONG MEMBERS INTO THE ORGANIZATION?

STRONG CAPACITY INFORMATION / AGREEMENTS AMONG MEMBERS ☐   AGREEMENTS BUT REGULAR MECHANISMS ☐   DEFICIENT CAPACITY / MINORITY AGREEMENTS ☐

CONTRIBUTION FISHER´S KNOWLEDGE

3.2 HOW IS YOUR GENERAL KNOWLEDGE ABOUT FISHING RESOURCES AND ITS ENVIRONMENT LINKED TO MANAGEMENT AREAS?

CONSIDERABLE ☐   ENOUGH ☐   SOMETHING SUFFICIENT ☐   LITTLE ☐   VERY LITTLE ☐

CHANGE RATE IN THE NUMBER OF MEMBERS

3.3 IN THE LAST 10 YEARS, HOW THE MEMBERS NUMBER HAVE VARIED IN YOUR ORGANIZATION?
(relate the number of currently participants, compared to previous years)

INCREASED (proportion > 1) ☐   SAME (proportion = 1) ☐   DECREASED (propotion < 1) ☐

INTERACTION AT LEADERS LEVEL

3.4 DO YOU HAVE KNOWLEDGE ABOUT YOUR LEADERS PARTICIPATION IN OTHER GREMIAL INSTANCES (such as federations, confederations, and so on)?

If the answer is **POSITIVE**,

PLAYS ROLE IN SEVERAL INSTANCES ☐   PLAYS ROLE IN ONE INSTANCE ☐   IT DOESN'T MEET MANAGERIAL ROLES ☐

GENDER ROLE (*)

3.5 IN THE LAST YEARS, HOW OFTEN DO **WOMEN PARTICIPATE AS MEMBER** IN THE ORGANIZATION?

VERY FREQUENT ☐   FREQUENT ☐   OCCASIONALLY ☐

RARELY ☐   NEVER ☐

3.6 BY OTHER SIDE, HOW DO YOU CLASSIFY **WOMEN´S PERFORMANCE** IN THE ORGANIZATION?

VERY GOOD ☐ GOOD ☐ REGULAR ☐ BAD ☐ VERY BAD ☐

Details:

SECTION IV: Ethic indicator

CULTURAL VALUE

4.1 IN YOUR OPINION, WHAT DO YOU EXPECT FROM THIS OFFICE LINKED TO OWN ACTIVITY FROM MEABR FOR THE FUTURE GENERATIONS?

CULTURAL IDENTITY AND DIVERSIFY ☐ ONLY AS AN **ALTERNATIVE** ACTIVITY ☐ YOUNG PEOPLE **DO NOT FIND IT AS AN ATRACTIVE** JOB ☐

RIGHT MANAGEMENT

4.2 ACCORDING YOUR PERCEPTION, DECISION-MAKING IN ALL THE SYSTEM. HOW IT HAS BEEN CARRIED OUT IN THE LAST YEARS?
(e.g., how make-decision about the fishing? and so on)

EQUALLY PARTICIPATION INCLUDING FISHERS ☐ GOVERMENT WITH SCIENTIFIC ADVISING ☐ ONLY GOVERNMENT ☐

EVOLUTION DESTRUCTION ECOSYSTEM

4.3 HOW THE NUMBER OF INDUSTRIAL ESTABLISHMENTS HAS EVOLVED NEAR TO MEABR?
(+)
NO INCREASED / WASTE CONTROL ☐ INCREASE / WASTE CONTROL ☐ NO INCREASED / POOR CONTROL ☐

INCREASED / ZERO CONTROL ☐ POLLUTION ☐
(-)

VULNERABILITY TO OUTSIDERS

4.4 HOW FREQUENT IS THE ENTRANCE OF OUTSIDER BOATS (INCLUDE DIVERS) TO MEABR?

NULL ☐ REGULAR ☐ FREQUENTLY (VULNERABLE TO OUTSIDERS' ENTRANCE) ☐

EVOLUTION OF ILLEGAL FISHING (POACHING)

4.5 HOW DO YOU CATEGORIZE **ILLEGAL FISHING** IN THE MEABR?. This includes several illicit activities: Fishing without permission, don´t respect to catch quotas, don´t declare or give false information about harvest among others.

NONE ☐ SOME, HAVE RULES TO REDUCE POACHING ☐ TOO MUCH (ILLEGAL FISHING) ☐

DAMAGE MITIGATION PROGRAM

4.6 IS THERE ANY MANAGEMENT PLAN TO MINIMIZE NEGATIVES ENVIROMENTAL IMPACTS THAT OCCUR INTO MEABR?

YES ☐ NO ☐

4.7 If it is **AFFIRMATIVE**, HOW WAS IT CARRIED OUT?

EFFECTIVE / IMPLEMENTATION PROGRAM ☐ SOME OF MITIGATION MEASUREMENT ☐ NONE ☐

SECTION V: Economic indicator

COST PER KILO OR UNIT

(ask if you have the information, otherwise the shipowner)

5.1 DO YOU BRING A REGISTER OF EXPENSES? YES ☐ NO ☐

If the answer is **AFFIRMATIVE**, request totals by item
ON THE CONTRARY, ASK FOR TICKETS AND PROOF OF EXPENCES TO OBTAIN DATA AT THE NEXT VISIT, IN ___________DAYS.
(For both options, in the case of do not account tickets, consider what you buy from "a lo amigo")

COST OF OPERATION

in what expend?
how much expend in each item?

item	quantity	price unit ($)	annual frequency
outboard engine mix			
compressor fuel			
boat supplies			
talc diving suit			
other expenses			

MAINTENANCE COST

in what expend?
how often?

item	quantity	price unit ($)	annual frequency
outboard engine			
compressor			
careening			
repair diving suits			
replacement diving suits			
mechanical maintenance			
other expenses			

*other expenses: motor oil, etc.

COST-BENEFIT

5.2 ACCORDING TO COSTS (OPERATIVE AND VARIABLE) THAT GENERATE IN THE MEABR, HOW DO YOU CLASSIFY (PROPORTION= Benefit/Cost)?

MEABR GENERATES PROFIT (proportion B/C > 1) ☐ IT HAS REMAINED STABLE ☐ MEABR GENERATES LOSSES (proportion B/C < 1) ☐

INDIRECT TOTAL INCOME

5.3 HOW DO YOU CLASSIFY THE **FISHING ACTIVITY IN THE MEABR** RESPECT TO OTHER ACTIVITIES CARRIED OUT BY THE ORGANIZATION?

MAIN ACTIVITY ☐ COMPLEMENTARY ACTIVITY ☐ MARGINAL ACTIVITY ☐

comments:

DEBT LEVEL

5.4 DO YOU KNOW ABOUT **SOME DEBT THAT YOUR ORGANIZATION** HAS WITH FINANCIAL ENTITIES? YES ☐ NO ☐

If the answer is **AFFIRMATIVE**, continue with 5.5 approximate average amount of debt (optional) $

5.5 IN THE LAST 5 YEARS, HOW DO YOU CATEGORIZE THE DEBT LEVEL (=Annual debt/Total income)?

OPTIMAL DEBT LEVEL (<40%) ☐ RISKY DEBT LEVEL (> 40%) ☐

SECTION VI: Institucional indicator

PRESENCE AND ADVICE

6.1 INDICATE, WHICH ENTITIES ACTIVELY PARTICIPATE OR SUPPORT IN THE SURVEILLANCE OF YOUR MEABR?

SERNAPESCA ☐ COAST GUARD ☐ PRESENCE OF SOME AUTHORITY (POLICE) ☐ NOTHING ☐

INTERNAL CONFLICT LEVEL

6.2 HOW DO YOU QUALIFY THE AMOUNT OF CONFLICTS GENERATED INTO YOUR ORGANIZATION?

VERY FREQUENT (> 60%) ☐ REGULAR (BETWEEN 40% AND 60%) ☐ NULL (<= 30%) ☐

6.3 INDICATE THE FOLLOWING:

No. of sanctions ☐

In how long time? ☐

detail, WHAT KIND OF **SANCTIONS** HAVE BEEN APPLIED?

EXTERNAL CONFLICTS LEVEL

6.4 HOW DOES THE AMOUNT OF CONFLICTS GENERATED OUTSIDE YOUR ORGANIZATION? (e.g., neighbors of the organization, public, and private actors)

HIGH (> 3 ORGANIZATIONS OR INSTITUTIONS) ☐ MEDIUM (BETWEEN 1 AND 2) ☐ LOW (< 1) ☐

6.5 LIST, WHAT KIND OF CONFLICTS HAVE BEEN GENERATED?

CONFLICT RESOLVE MECHANISM

6.6 ARE THERE ANY MECHANISMS TO RESOLVE CONFLICTS INTO YOUR ORGANIZATION?

YES, VERY EFECTIVE ☐ EXISTS, BUT IT ISN´T EFFECTIVE ☐ NULL ☐

6.7 DETAIL, WHAT ARE THESE MECHANISMS? IF NOT EXIST, WHY REASONS?

6.8 ARE THERE ANY MECHANISMS TO RESOLVE CONFLICTS OUTSIDE YOUR ORGANIZATION?

YES, VERY EFECTIVE ☐ EXISTS, BUT IT ISN'T EFFECTIVE ☐ NULL ☐

6.9 DETAIL, WHAT ARE THESE MECHANISMS? IF NOT EXIST, WHY REASONS?

COMPLIANCE OBJECTIVES LEVEL

6.10 ACCORDING YOUR OPINION, DOES YOUR ORGANIZATION HAVE **ANNUAL OBJECTIVES**? (e.g., projects and others)

YES ☐ NO ☐

If it is **AFFIRMATIVE**, WHAT IS **THE LEVEL OF FULFILLMENT** ABOUT SUCH **OBJECTIVES**?

MORE THAN 60% ☐ BETWEEN 40% AND 60% ☐ LESS THAN 30% ☐

No. OF FUND PROJECTS

6.11 IS THERE ANY CO-FINANCIAL SUPPORT FOR PROJECTS IN YOUR ORGANIZATION?

YES ☐ NO ☐

IF it is AFFIRMATIVE, HOW MANY **PROJECTS HAVE BEEN EXECUTED** IN THE LAST 5 YEARS?

MORE THAN ONE PROJECT ☐ EQUALS A ONE PROJECT ☐ NONE ☐
