# Supplementary material for: Integrating human and ecological dimensions: The importance of stakeholders’ perceptions and participation on the performance of fisheries co-management in Chile
Source: PLoS One. 2021 Aug 11;16(8):e0254727. doi: 10.1371/journal.pone.0254727 (PMC8357100; doi:10.1371/journal.pone.0254727)
Supplement: S3 Appendix — (PDF) [file pone.0254727.s010.pdf]

S3 Appendix. Questionnaire for non-fishers (decision-makers, academics and consultants)

BLOCK 1

Age: ______ Sex: Male ______ Female ______

Role: __________________________ You belong to: ____________________________________ (academy, government, private sector, others)

BLOCK 2

Indicate how each of the following ecological-fishery and human components is currently affecting the future of MEABR in Biobio Region, Chile. With value zero (0) are the worst scenario and ten (10) the best-case scenario (mark with an X your answer).

		**unfavorable**				**regular**			**very favorable**			
		**0**	**1**	**2**	**3**	**4**	**5**	**6**	**7**	**8**	**9**	**10**
**Technological**	1. Fleet capacity evolution (number of boats and **divers**) into MEABRs of the region, compared to 10 years ago.											
	2. Length and tonnage in GRT[1] of the vessel participating in the MEABR compared to 10 years ago.											
	3. The use of new fishing gear in the management area in the last ten years.											
	4. The distance (km or miles) traveled, the time (hours) it takes for the boat to reach the MEABR.											
	5. Reduction in harvest intensity (harvest/total number of divers) in the MEABR.											
	6. The surveillance system is applied in the management area to minimize poaching.											
	7. Other activities that have side effects on the MEABR.											
**Economic**	1. Harvest proportion used concerning authorized quotas for species harvested in MEABR.											
	2. Expenses generated by the fishing activity during the "harvest season" that influence the cost.											
	3. Harvest profits evolution compared to 10 years ago.											
	4. The income generated by harvesting during the last ten years, compared to other activities.											
	5. The profits distribution between the shipowner and other participants in the harvest season.											
	6. Income from other activities.											
	7. Subsidies received and the purpose for which they used during the last ten years.											
	8. The fisher organization's capacity in response to debts owed to financial institutions and other entities.											
	9. The total income of fisher in respect to minimum wage ($301,000).											
	10. The extent of sales of harvested species from the MEABR, where the product goes to.											
**Institutional**	1. Policy presence and advising with MEABR activities.											
	2. Current collaboration in the right management as scientists, politicians, and fishers.											
	3. Sanctions applied inside the fisher organization.											
	4. Mechanisms to resolve conflicts with external agents: AFOs[2], institutions, or others.											
	5. Networks that the fisher organization has with institutions to obtain benefits.											
	6. Networks generated between fisher organizations to commercial strategies and surveillance of MEABRs.											
	7. Fulfillment of objectives associated with the work plans by fisher organizations.											
	8. The number of projects linked to the development of MEABR.											

1) GRT=gross registered tonnage; 2) AFOs= artisanal fisher organizations

		unfavorable				regular			very favorable			
		0	1	2	3	4	5	6	7	8	9	10
**Social**	1. Exchange of information for management and decision-making within the fisher organization.											
	2. Consideration of fishers' knowledge about fishery resources and the marine environment.											
	3. Members' participation in meetings, monitoring study, and sustainable practices.											
	4. The number of members evolution in the fisher organization during the last ten years.											
	5. Representation of the organization's leader in other trade associations.											
	6. Dynamics in the replacement of leaders within the fisher organization.											
	7. Educational level of fishers (members and leaders) belongs to the organization.											
	8. Tenure of the MEABR, its continuity in the monitoring studies and harvests.											
	9. Development level of fishing coves in the Biobio Region.											
	10. Women's participation within the fisher organization.											
	11. Women's performance as members of the fisher organization.											
**Ethical**	1. Relative prestige of the MEABR activity in comparison to other activities.											
	2. The accessibility for the activity according to its location.											
	3. Presence of fisher in the governance and management of MEABR.											
	4. Evolution of the number of industrial establishments, among others close to MEABR.											
	5. Entrance of outsider boats (including divers) into the MEABR.											
	6. Evolution of illegal fishing within the MEABR during last years.											
	7. Management plan to minimize negatives environmental impacts that occur in MEABR.											
**Ecological-fishery**	1. The amount of harvesting done compared to 10 years ago.											
	2. Modifications of the habitable area according to target species harvested into MEABR.											
	3. The number of target species harvested below commercial size.											
	4. Evolution of the condition (weight and abundance) of target species harvested in MEABR.											
	5. Evolution of density levels (no. of individuals/m^2) of target species in the MEABR.											
	6. Compliance with harvest quota for Chilean abalone 'loco' or other target species in the management area.											
	7. The difference between the abundance of target species harvested today and ten years ago.											

BLOCK 3

Indicate the importance of the following ecological-fishery and human components for the state of MEABR in Biobio Region, Chile. With value zero (0) are indicating less important and ten (10) very important (Mark with an X your answer).

		less important					regular					very important
		0	1	2	3	4	5	6	7	8	9	10
**Technological**	1. Fleet capacity (number of boats and **active divers**).											
	2. Length and tonnage evolution of boats, which participates in the harvest.											
	3. Use of new fishing gear within the management area.											
	4. The distance traveled, the time it takes for a boat to reach the MEABR.											
	5. No. of divers currently working in MEABR concerning the harvesting of target species.											
	6. Monitoring and surveillance are applied in the MEABR to minimize poaching.											
	7. Other activities that interfere with the management area.											
**Economic**	1. Harvest proportion used concerning authorized quotas.											
	2. The expense generated during the harvest season.											
	3. The benefit of fishing activity (harvest) in the MEABR.											
	4. The income generated by harvest concerning other activities carried out by members.											
	5. Equality in the distribution of benefits during harvest season.											
	6. Income from other activities (other than fishing).											
	7. The subsidies received from the artisanal fishing sector.											
	8. Debts owed by the fisher organization to financial entities.											
	9. The total income in fishers' households concerning the income received by other activities.											
	10. The market where the harvested target species is sold (local, region, country, or export).											
**Institutional**	1. Presence and support of government authorities in the MEABR performance.											
	2. Integration of the different stakeholders in the management of MEABR.											
	3. Sanctions applied within the fisher organization.											
	4. Mechanisms to resolve conflicts generated in and out of the fisher organization.											
	5. Interaction of the fisher organization with other institutions to obtain benefits.											
	6. Networking among fisher organizations to share benefits.											
	7. Fulfillment of objectives associated with the work plans by fisher organizations.											
	8. The number of projects linked to improving the MEABR.											

		less important				regular						very important
		0	1	2	3	4	5	6	7	8	9	10
Social	1. Exchange of information between participants in the fisher organization (leaders and members).											
	2. Evaluation of the fisher knowledge input into decision making.											
	3. Presence of fisher in decision-making related to management areas.											
	4. The number of members into fisher organization.											
	5. Role of the leader in other instances: federations, confederations, among others.											
	6. Frequency on leaders replacement into fisher organization.											
	7. Average educational level of members that influences the MEABR performance.											
	8. Tenure of the MEABR, its continuity in the monitoring studies and harvests.											
	9. Development level of fishing coves in the region.											
	10. Participation of women into the fisher organization with MEABR.											
	11. Presence of women in leadership roles and management activities related to MEABR.											
Ethical	1. Artisanal fishing activity as a desirable way of life for future generations.											
	2. The accessibility for the activity according to its location.											
	3. Presence of fisher in the governance and management of MEABR.											
	4. Evolution of the destruction of the marine ecosystem, e.g., industrial establishments.											
	5. Vulnerability to ouside boats in the management area.											
	6. Evolution of illegal fishing in recent years.											
	7. Mitigation plans to minimize environmental impacts in the surroundings of the MEABR.											
Ecological-fishery	1. Productive level (abundance) of the target species extracted in the MEABR.											
	2. The amount of Chilean abalone 'loco' or other target species harvested before maturity.											
	3. Evolution of the condition (weight and abundance) of target species harvested in the last ten years.											
	4. Evolution of density levels of target species in the last ten years.											
	5. Reduction of the habitable area of target species harvested in the MEABR.											
	6. The level of compliance with the harvest of target species in the last ten years.											
	7. Number of target species harvested.											
	8. Change in target species harvested compared to 10 years ago.											
