# Supplementary material for: Integrating human and ecological dimensions: The importance of stakeholders’ perceptions and participation on the performance of fisheries co-management in Chile
Source: PLoS One. 2021 Aug 11;16(8):e0254727. doi: 10.1371/journal.pone.0254727 (PMC8357100; doi:10.1371/journal.pone.0254727)
Supplement: S1 Text — (DOCX) [file pone.0254727.s011.docx]

**S1 Text**

**Selection, stratification and sampling design**

**First stage.** We selected those MEABRs which meet the following criteria: (i) active with continuous monitoring studies in the last 12 years; (ii) the main exploited species is the Chilean abalone ‘loco’ (*C. concholepas*) ; and (iii) with harvests approved by at least 6 monitoring studies. From 78 MEABRs approved and awarded in Biobio region ^[[1]](#footnote-1)^, only 21 MEABRs met these criteria.

**Second stage.** Due to the heterogeneity of the 21 MEABRs selected and their artisanal fishers’ organizations (AFOs), they were grouped according to three variables: (i) yield, which is the harvest per effective area (0.28 – 23.29 t/m^2^*1000), (ii) suitable substrate (63.30 – 958.86 m^2^/1000), and (iii) and available area per member (0.07 – 2.55 m^2^/member*10000). We applied a cluster analysis method to the MEARBs vs. variables matrix. First, a hierarchical method estimated the number of possible adjustments by group. Then, a second method called portioning method or k-means algorithm ^[[2]](#footnote-2)^ selected several appropriate settings, resulting in three groups of MEABRs or strata. These strata were used for estimating the sample size per strata. We performed computations using the R statistical language ^[[3]](#footnote-3)^, and we conducted the Cluster analysis using the factoextra library ^[[4]](#footnote-4)^.

**Third stage.** Sample size was estimated using proportional stratified sampling. From the population size of fishers ($N$ = 1070), sizes of population strata ($N_{1}$, $N_{2}$, …, $N_{h}$) were obtained according to the grouping considered in the second stage. From these population strata, sample sizes of each strata were calculated ($n_{1}$, $n_{2}$, ..., $n_{h}$). The sample sizes of each strata were proportional to the sizes of population strata (AFOs and fishers) according to the following formula:

$n_{h}=n\times\frac{N_{h}}{N}$ (1)

where $n_{h}$ is the sample size of h-th stratum, $n$ is the sample size, $N_{h}$ is the population size of h-th stratum, and $N$ is the population size. The sample size of respondents (leaders and members) was calculated using the following formula:

$n=\frac{{Z_{\propto}}^{2}\times p\times q\times N}{e^{2}\left( N-1 \right)+{Z_{\propto}}^{2}\times p\times q}$ (2)

where $n$ is the sample size, $Z$ is the standard normal quantile at 95% probability (i.e. set equal to 1.96), $N$ is the population size, $e$ is a sample error of 10%, $p$ and $q$ represent the sample proportion (binomial distribution), set as $p=q=0.5$. This resulted in a sample size of 88.21 respondents. To consider potential non-responses and non-useable responses, we added 50% to the sample size of 88 respondents, resulting in a final sample size of 132 fishers. Finally, the sample size of each stratum in number of fishers is shown in Table 2 (this paper).

Although most AFOs possess a single MEABR, among the 21 MEABRs selected in this study, there were three AFOs possessing two MEABRs each. These management areas were: “Sindicato de Trabajadores Independientes, Pescadores Artesanales, Buzos Mariscadores y Actividades Conexas de Caleta Punta Lavapie”, “Cooperativa de Pescadores Pelilleros, Isla Santa Maria Limitada”, and “Organización Comunitaria Funcional de Pescadores Artesanales y Buzos Mariscadores de Isla Mocha”. As a final stage, we decided to replace these with three additional MEABRs: two were related to seaweed harvesting (these are bahc=Rari and golc=Pueblo Hundido), and the third one was venturing into small-scale aquaculture as one of its extra activities (gold=Laraquete MEABR sector). In this way, the number of 21 MEABRs was maintained with 18 corresponding AFOs.

**Flowchart of selection, stratification and sampling design**


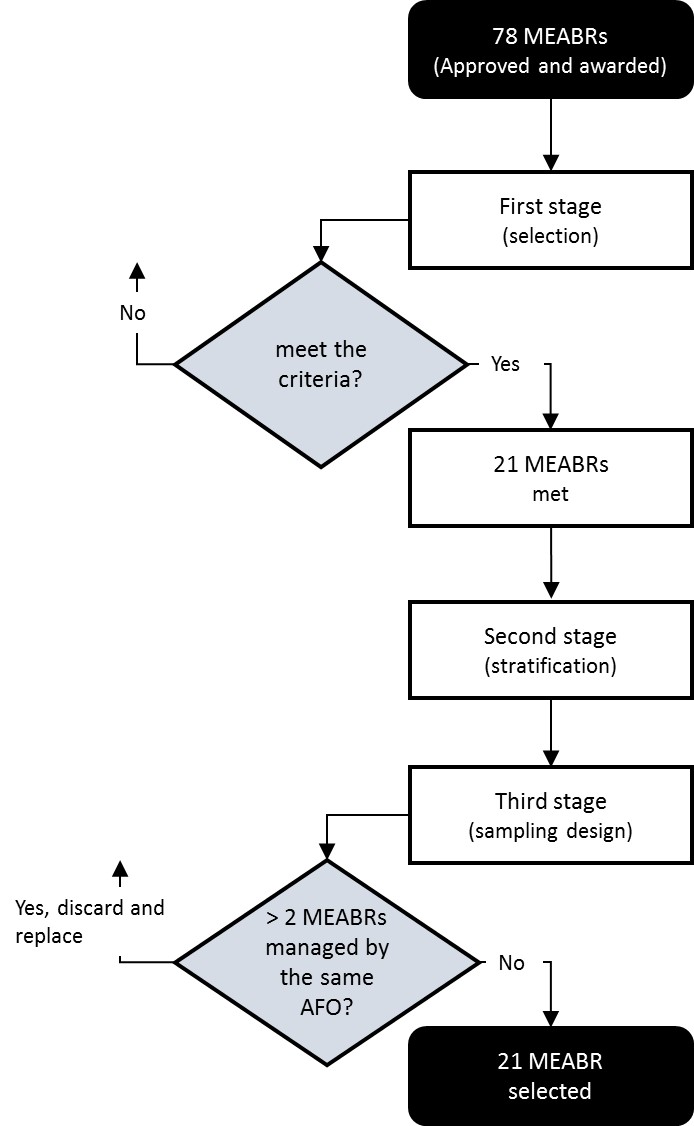


1. SUBPESCA (The Undersecretariat for Fisheries and Aquaculture), ‘Estado de Tramitación AMERB (Cobertura Geográfica). AMERB ESTADOS AMERB_ENERO_2018_USUARIOS.Kmz.’, 2018 <http://www.subpesca.cl/portal/619/w3-article-79986.html>. [↑](#footnote-ref-1)
2. Alboukadel Kassambara, ‘Practical Guide to Cluster Analysis in R: Unsupervised Machine Learning (Vol. 1): STHDA’, 2017 <http://www.sthda.com/english/articles/25-clusteranalysis-in-r-practical-guide/>. [↑](#footnote-ref-2)
3. R Core Team, ‘R: A Language and Environment for Statistical Computing. Version 3.6.1. R Foundation for Statistical Computing, Vienna.’, 2019 <http://www.r-project.org>. [↑](#footnote-ref-3)
4. Alboukadel Kassambara, ‘Factoextra: Extract and Visualize the Results of Multivariate Data Analyses. R Package’, 2020 <http://www.sthda.com/english/rpkgs/factoextra>. [↑](#footnote-ref-4)
