# Supplementary material for: Integrating human and ecological dimensions: The importance of stakeholders’ perceptions and participation on the performance of fisheries co-management in Chile
Source: PLoS One. 2021 Aug 11;16(8):e0254727. doi: 10.1371/journal.pone.0254727 (PMC8357100; doi:10.1371/journal.pone.0254727)
Supplement: S2 Text — (DOCX) [file pone.0254727.s012.docx]

**S2 Text**

**Some details about payments**

The payment is not regular. Most fishers work in the traditional artisanal fishery of sardine, anchovy, hake, and jumbo squid, as well as in other activities related to farming, livestock, and tourism.
