# Supplementary material for: Integrating human and ecological dimensions: The importance of stakeholders’ perceptions and participation on the performance of fisheries co-management in Chile
Source: PLoS One. 2021 Aug 11;16(8):e0254727. doi: 10.1371/journal.pone.0254727 (PMC8357100; doi:10.1371/journal.pone.0254727)
Supplement: Attachment — Submitted filename: Response to Reviewers.docx [file pone.0254727.s013.docx]

**Editor-in-Chief,**

*Journal of PLOS ONE*

RE: PLOS ONE Decision: Revision required [PONE-D-20-30809] - [EMID:a380eddb68b73dd6]

Below, we respond (in blue text) to the editor’s and reviewer’s comments.

Original comments from the Editor

I tend to agree with the comments and suggestions made by reviewer #1, the manuscript needs to be re-casted following the steps 1-3 described by reviewer #1, and in the introduction you need to clearly explain the needs and provide evidence for such study as well as the theoretical background with the respective cited references.

Authors’ response to the original comment from the Commissioning Editor

We would like to thank the Editor-in-Chief and the reviewer #1 for providing critical comments of our research. We have given careful consideration to each comment, resulting in a thorough revision of our text and several key modifications, including:

Introduction has been rewritten regarding the theoretical background of the research. Moreover, we have modified the abstract which justify why there is a need for our study.

The addition of citations of recent, relevant, and related research. These added references directly address the comments of Reviewer #1.

Several sentences and sections have been rewritten and/or include new information, methods, results, discussion, conclusion and references. Also, we carried out an editorial assistance to improve the English. These changes address the comments of Reviewer #1.

We consider that our revised manuscript has been greatly improved in terms of overall quality, adequately responding to all Editor and Reviewer concerns. Please find our detailed replies to reviewer below.

Reviewers’ comments

Reviewer #1

This manuscript provides an interesting study of a necessary issue: how to incorporate the social dimension of marine resource management. The authors provide a well-rounded case study in Chile. However, the study has some important methodological shortcoming. Moreover, is poorly written which makes it very challenging to judge its merit. I would recommend authors to seek editorial assistance before re-submitting this article. I provide some overall comments and then some specific comments.

Response: Thanks for the valuable comment. We have improved the manuscript by rewriting several sentences and sections. We have also added new information, discussion, and references to improve the overall quality of our review. Finally, a native English speaker revised the entire manuscript to ensure grammatical correctness and clarity for the intended audience.

1) There is an absence of a theoretical background in the introduction (this is clearly reflected in the abstract which does not contain a single line referring to why there is a need for such a study). Understanding the perception of those participating in fisheries is clearly important and this is vaguely stated in the first two paragraphs. But there has been extensive work looking at how perceptions affect performance in fisheries settings, which the authors do not reference. I would encourage authors to re-write the introduction including theoretical perspectives on what they are measuring (e.g. role of gender). Otherwise, it becomes quite challenging for the reader to understand why you did the analysis you did.

Response: According to the first point, we have now re-written the new introduction and modified the abstract which justify why there is a need for our study. We add references related to theorical background about perceptions on the performance of fisheries communities involving participation and role of gender.

2) The methodological approach has some important shortcoming that need to be reconsidered. The grouping strategy is very unclear, not only in how authors did it, but also on why. Moreover, the sample size calculation is misleading as they only consider the overall population of fishers, when their design is really stratified into MEARBs. I provide more details on this below.

Response: We thank for highlighting this methodological issue. We reviewed it again, made corrections, modifications and now there is a description of the grouping strategy of the MEABRs in the supplementary material (S1 Text).

3) Overall, the manuscript is very poorly written. There are sections that don’t seem to have been edited at all (e.g. lines 67-78: “Several fisheries worldwide are now using TURFs as a management strategy in several fisheries around the world”) while others are very unclear, and the English needs to be polished. I provide several examples in the specific comments.

Response: We have improved the manuscript by rewriting several sentences and sections. The English has been polished along the full manuscript.

Specific comments

Introduction

- Paragraph 38-47: I think this paragraph could be re-written to more clearly make the point you are trying to make. Are you trying to say that there is a threat of overfishing that needs to be considered? In which fleets? Does the focus need to be put in the social realm of fisheries management?

Response: Thanks for the valuable comment. We clarified this in the paragraph #1 of the new introduction (now lines 44-50).

“The purpose of fisheries management is to preserve and ensure sustainable use of the resource (1). However it is still necessary to improve the performance of fisheries where there are serious problems of overfishing and the management system is deficient (2). For this, several authors have suggested that integral monitoring should be improved for all fisheries, including artisanal fisheries (3,4). As fisheries are complex socio-ecological systems (SES), the interaction between the ecological components and the social realm that make up these systems needs to be strengthened (5,6)”.

- Line 51-53, which impacts?

Response: This line corresponds to paragraph #2 of the new introduction (now lines 54-57).

“Nevertheless, data availability for artisanal fisheries is poor and it is therefore difficult to understand and effectively manage such fisheries, which are also poorly regulated (4). In addition, the drive for short-term gains generates fishing impacts such as resource depletion and loss of biodiversity (9)”.

- Line 51, it is not clear what the authors mean by “weaknesses in our knowledge of human responses”

Response: That line was replaced by the paragraph #3 of the new introduction (now lines 58-67).

“Consequently, the current dilemma facing fishery management is not just the limited understanding of the fish stocks or external pressures, but also knowledge of the human dimension and how it fits into fishery management (10–13). There is a broad theoretical background for the role of the human dimension in fishery co-management (10,14–19) but it is necessary to test this empirically with real fishing communities in order to understand and improve sustainable fisheries (20–22). The Code of Conduct for Responsible Fisheries (CCRF, article 12) underlines the need to investigate and plan fishery measures, including social, legal and institutional aspects (23). In other words, it is not possible to manage a fishery without managing the fishers, as the activity faces a diversity of problems which require holistic strategies to guarantee its sustainability (24–28)”.

- Line 53-55 Provide references

Response: We have added new references to improve the overall quality of our review. This line now corresponds to second and third paragraph of the new introduction and respective references are provided.

For the paragraph #2 (now lines 51-57) are: Andreu-Cazenave et al. 2017 (4); Rousseau et al. 2019 (7); De Pádua Andrade and Schiavetti 2015 (8); Stafford 2019 (9).

And for the paragraph #3 (now lines 58-67) are: Ostrom 2009 (10); Espinoza-Tenorio et al. 2013 (11); Hernández, Segado, and Pitcher 2016 (12); van Putten et al. 2019 (13); De Young, Charles, and Hjort 2008 (14); Curtin and Prellezo 2010 (15); Fulton et al. 2011 (16); Valdés-Pizzini, García-Quijano, and Schärer-Umpierre 2012 (17); Reineman, and Kittinger 2013 (18); Arias-Schreiber, Wingren, and Linke 2020 (19); Koehn, Burgess et al. 2020 (20); Weber, Borit, and Aschan 2019 (21); Castrejón and Charles 2020 (22); FAO 1995 (23); Hall-Arber, Pomeroy, and Conway 2009 (24); van Putten et al. 2013 (25); Gelcich and O’Keeffe 2016 (26); Benson and Stephenson 2018 (27); Machado et al. 2019 (28).

- Line 53-55 authors already state something similar in the first paragraph. Both these paragraphs need a deep revision, as to make clear what is the message. As written, it is very difficult to understand the introduction.

Response: These lines have been replaced by the second (now lines 51-57) and third (now lines 58-67) paragraphs of the new introduction, making clear the need to investigate and plan the measures used, including social, legal and institutional aspects.

- Line 60: “Avoid marine resource issue” please re-write

Response: This line was replaced by the paragraph #4 of the new introduction (now lines 68-72).

“Given this reality, various studies have shown that one of the strategies for improving management and sustainability of coastal fisheries is the Territorial Use Rights for Fisheries or TURF system (29–35). The TURFs are designed to assign exclusive rights and privileges to certain user groups in specific areas to ensure the rational use of resources (36–38)”.

- Lines 60-66: The text could be improved substantially if authors better explain this system. A few notes: what do you mean by “set the rules …for fishers performance”?, in ii) authors sort of contradict themselves with i)….who sets the guidelines? (and what do they mean by guidelines?), government is responsible for enforcement, not compliance, iii) is unclear (how can only fishers execute co-management, which involved the government?)

Response: These lines were replaced by the paragraph #4 of the new introduction (now lines 68-78).

“... Unfortunately, for a variety of reasons some TURFs have never produced the results expected, which are: i) resource conservation and protection, ii) fair distribution of the benefits obtained, (iii) maintaining social cohesion and preserving cultural values and iv) scarce administrative, human and financial resources for surveillance of illegal fishing, among others (39,40). This is without doubt the main problem affecting much of the TURF system in Chile, also popularly known as the Management and Exploitation Areas for Benthic Resources or MEABR system”.

- Lines 72-73: are those studies focusing on social aspects or the AMERB themselves. Very unclear

Response: We have added new information and references to improve the overall quality of our introduction.

Paragraph #5 (now lines 79-92)

“The MEABRs gained traction in 1990 and over the years have been applied in a considerable geographic area. Despite some positive results both for environmental sustainability (41–43) and socio-economic effects (42,44–50), their management is still bedeviled by inefficiencies, because once a right is established, it is difficult to reverse or change it, even if unwanted results occur (48,51,52). For example, some MEARBs managing surf clams (53,54) could not solve the problems caused by the high temporal variability of this resource, and as a result, the institution of governance was abandoned and did not recover even when the resource itself recovered (48,51,55–60). Nevertheless, one of the favorable effects of the MEABRs has been the empowerment of fishers’ organizations, which have promoted collective actions among users (55,61). Collective action is broadly understood as “people joining together purposively for a common cause” (62), although collective action can be collaborative but also confrontational, depending on the relationship among stakeholders (63). Ever since their inception therefore, MEABRs have been adapted to the requirements of new regulations and fishing practices (64)”.

Paragraph #6 (now lines 93-103)

“The Biobio region in south-central Chile, is an example of how most of the results envisaged for the MEABR system have not been positive (65). This lack of positive results has in many cases caused frustration and the abandonment of MEABRs administered by artisanal fishers’ organizations (66–68). Management of the MEABRs is complex and the responses of fishers can have unforeseen consequences leading to failure. As stated earlier, there are many examples of the MEABR system being applied with no consideration of the socio-ecological systems upon which they were imposed (48,51,55,69). For that reason, finding out why the regulations have not been followed or respected could help to avoid the negative results the system has generated so far; and could explain how the lack of governance that prioritizes participatory management has contributed to the overexploitation and poor state of small-scale marine fishing worldwide (70)”.

- Lines 76-77: Durvillaea is not bull kelp

Response: This line was replaced and corresponds to paragraph #5 of the new introduction (now lines 79-92). We rectified that *Durvillaea antarctica* is commonly known as ‘cochayuyo’, is a large, robust southern bull kelp species.

- Line 79-81: References?

Response: The required references have been included in the new introduction.

Paragraph #7 (now lines 104-114)

“Under these circumstances, understanding how fishers perceive and use the resources will have important repercussions for the management and local policies of the MEABRs. This will also have a bearing on how to reduce conflicts and strengthen social networks within the context of a more comprehensive planning of the system. One recent work (71) concluded that if fishers’ organizations and consultants take as little part in the collective decision-making process as they have so far, it is unlikely that the necessary adjustments will be made to the system. Indeed, the different viewpoints of more men and women regarding management should be taken into account, in order to obtain a better understanding of the social interaction of women in an activity traditionally considered to be male-dominated (57,72–74). That's why women also need to have an increased right to participate in decision-making processes (75)”.

Paragraph #8 (now lines 115-122)

“Indeed, the perceptions of stakeholders (including fishers) are important indicators of the performance of the MEABR system, particularly when governance is deficient (76,77). Thus, for many of the world’s fisheries, including the Chilean exclusive access regime, a more holistic evaluation of the ecological and human dimensions is necessary (27,64,78–82). However, as far as we know, few studies in the region have systematically documented the perceptions of fishers in a socio-ecological context. Thus “each coastal territory and the fishers who live in it are heterogeneous, consequently although changes may be seen as transverse, no generalizations can be made regarding the local responses” ((73) page. 177)”.

- Line 82: Can you expand on this?

Response: We modified and expand this paragraph (now lines 79-86).

“The MEABRs gained traction in 1990 and over the years have been applied in a considerable geographic area. Despite some positive results both for environmental sustainability (41–43) and socio-economic effects (42,44–50), their management is still bedeviled by inefficiencies, because once a right is established, it is difficult to reverse or change it, even if unwanted results occur (48,51,52). For example, some MEARBs managing surf clams (53,54) could not solve the problems caused by the high temporal variability of this resource, and as a result, the institution of governance was abandoned and did not recover even when the resource itself recovered (48,51,55–60)”.

- Line 84: you mean collective action?

Response: Thanks for the valuable comment. In new lines 86-89, we defined collective action.

“Nevertheless, one of the favorable effects of the MEABRs has been the empowerment of fishers’ organizations, which have promoted collective actions among users (55,61). Collective action is broadly understood as “people joining together purposively for a common cause” (62)”.

- Line 95-97: By the time the reader gets to this, there has been absolutely no background on the theoretical perspectives for hypothesising this. The paper presents background on the system itself, but not on previous work that links individual characteristics and behaviour. This is a very hot research topic and there are dozens of studies that deal with this. I would strongly encourage authors to do a deeper literature review and re-frame their hypothesis/research questions based on what others have done in the past. As written, the introduction only states that the human dimension of management needs to be considered. This is OK, but only as a starting point, it does not suffice as a theoretical background.

Response: Thanks for the valuable comment. We have added a theoretical background of our hypothesis, with references and a new information to improve the overall quality in the new introduction.

Paragraph #6 (now lines 93-103)

“The Biobio region in south-central Chile, is an example of how most of the results envisaged for the MEABR system have not been positive (65). This lack of positive results has in many cases caused frustration and the abandonment of MEABRs administered by artisanal fishers’ organizations (66–68). Management of the MEABRs is complex and the responses of fishers can have unforeseen consequences leading to failure. As stated earlier, there are many examples of the MEABR system being applied with no consideration of the socio-ecological systems upon which they were imposed (48,51,55,69). For that reason, finding out why the regulations have not been followed or respected could help to avoid the negative results the system has generated so far; and could explain how the lack of governance that prioritizes participatory management has contributed to the overexploitation and poor state of small-scale marine fishing worldwide (70)”.

Paragraph #7 (now lines 104-114)

“Under these circumstances, understanding how fishers perceive and use the resources will have important repercussions for the management and local policies of the MEABRs. This will also have a bearing on how to reduce conflicts and strengthen social networks within the context of a more comprehensive planning of the system. One recent work (71) concluded that if fishers’ organizations and consultants take as little part in the collective decision-making process as they have so far, it is unlikely that the necessary adjustments will be made to the system. Indeed, the different viewpoints of more men and women regarding management should be taken into account, in order to obtain a better understanding of the social interaction of women in an activity traditionally considered to be male-dominated (57,72–74). That's why women also need to have an increased right to participate in decision-making processes (75)”.

Paragraph #8 (now lines 115-122)

“Indeed, the perceptions of stakeholders (including fishers) are important indicators of the performance of the MEABR system, particularly when governance is deficient (76,77). Thus, for many of the world’s fisheries, including the Chilean exclusive access regime, a more holistic evaluation of the ecological and human dimensions is necessary (27,64,78–82). However, as far as we know, few studies in the region have systematically documented the perceptions of fishers in a socio-ecological context. Thus “each coastal territory and the fishers who live in it are heterogeneous, consequently although changes may be seen as transverse, no generalizations can be made regarding the local responses” ((73) page. 177)”.

Methods

- Line 116-118: Phrase not finished

Response: In the new lines 146-149, we have completed the phrase.

“Artisanal fishers are settled in both urban (54.3%) and rural (45.7%) localities (84), allowing them to develop their activities linked to fishing, seaweed collection and alternative activities such as commerce, tourism, agriculture and livestock among others”.

- Line 130: should be differences

Response: Done (new line 160).

- Line 130-134: Please explain this a bit more clearly and provide references. What do you mean by “accessibility had implications for its performance”?

Response: In new lines 165-168, we have explained and added references about accessibility.

“In addition, accessibility (e.g. those MEABR in isolated location or with easier access) has had implications on MEABR performance, as those MEABRs near urban centers have received more funding from governmental institutions (86), but are also more vulnerable to illegal fishing (4,67,87)”.

- Lines 135: Please explain what do you mean by decreed

Response: Now lines 182-186, we have clarified the terminology in the Table 1 footnote.

“Approved and awarded: those MEABRs requested by a fisher’s organization with an ESBA Proposal; Approved but not awarded: those MEABRs that are available to be used but to date no organization is interested, has expired or has been returned by a fisher’s organization, or is pending decision by competition among 2 or more organizations, Rejected: those MEABRs that did not get approval to be used because the process stopped earlier (Chavez et al. (90))”.

- Table 1, please fix last column

Response: The last column of Table 1 has been fixed.

- Line 149: Why were those included and not others?

Response: In lines 188-190, we added a new paragraph explaining the selection of MEABRs.

“From the 78 MEABRs approved and awarded, we selected 21 MEABRs according to the selection, stratification and sampling design described in the supplementary material (S1Text)”.

- Line 152: does that mean those are closed now?

Response: These MEABR are still operative at the present time. We have modified the information for better understanding (now lines 192-193).

“The fisheries statistical data about the selected MEABRs was collected over 18 years (between 2000 and 2017)”.

- Authors don’t explain why they have done this grouping and why they arbitrarily decided to replace some AMERB

Response: We have added the grouping strategy of the MEABRs in the supplementary material (S1 Text). In particular, we explain that: “we decided to replace these with three additional MEABRs: two were related to seaweed harvesting (these are bahc=Rari and golc=Pueblo Hundido), and the third one was venturing into small-scale aquaculture as one of its extra activities (gold=Laraquete MEABR sector). In this way, the number of 21 MEABRs was maintained with 18 corresponding AFOs”.

- Moreover, it is very unclear HOW this grouping was developed. Very unclear section.

Response: The grouping strategy of the MEABRs has been clarified the supplementary material (S1 Text) including a graphical scheme.

- Line 173, why do you consider a different number than what’s stated in Table 2? (1080 vs 1230)

Response: We thank the reviewer for highlighting this. It was a mistake on our behalf, and we have corrected some numbers in Table 2. We have now checked that the correct numbers are: 467 + 288 + 315 = 1070.

The details of calculations are included in the Selection, stratification and sampling design in supplementary material (S1 Text).

- Authors seem to ignore the fact that this this is a stratified sample, is not just one population, is a series of groups that you are interested in. As such, just aggregating and obtaining one sample size is not the appropriate way to do it if you want to say something about the differences between groups. Moreover, they seem to ignore the fact that there are different population sizes in each group.

Response: We now include in the methodological section details and description about the grouping strategy for the MEABRs, especially for those selecting in our research.

Following your comment, we reconsidered the sampling design described in the supplementary material (S1 Text). The sample sizes of each strata were proportional to the sizes of population strata (AFOs and fishers) according to the following formula:

$n_{h}=n*\frac{N_{h}}{N}$,

where $n_{h}$ is the sample size of h-th stratum, $n$ is the sample size, $N_{h}$ is the population size of h-th stratum, and $N$ is the population size.

- Line 190-191: Please describe in more detail and better justify the inclusion of decision-makers

Response: In new lines 218-228, we have justified the inclusion of decision-makers.

“We also surveyed decision-makers because this will show us a wider picture of the situation regarding MEABRs at both regional and national levels. The sample selection was not random, but ‘key informants’ were selected. The intentional or convenience sampling (91) was carried out by the first author. This sampling ensured that participants with well-known expertise were chosen. For this survey, 20 expert participants were selected from the academy, the Undersecretariat for Fisheries and Aquaculture (SUBPESCA), National Fisheries and Aquaculture Service (SERNAPESCA), Fisheries Development Institute (IFOP), and specialist consultants’ staff. Semi-structured interviews were conducted with the representatives of each institution. In addition, the questionnaire was applied to collect data about their perceptions and their experiences. The interviews were carried out between July and September 2019 and took approximately 1 hour”.

- Line 192-193: But how was these data analysed?

Response: In Data Analysis (new lines 257-261), we added the methods used to analyze decision-makers survey data.

“Questionnaire-based data obtained from decision-makers were ordinal data measuring different levels, from very positive to very negative. We explored the scorings from decision-makers and fisher’s perceptions separately and compared them. We applied the Likert scale to measure respondents’ attitudes to a particular question. We used the Likert package (95) in R software”.

- Line 196-197: Why include the central zone?

Response: In new lines 218-219, we explained that “We also surveyed decision-makers because this will show us a wider picture of the situation regarding MEABRs at both regional and national levels”.

That’s why, we have chosen some professional staff from the Biobio region and, also from the national central government (Valparaiso).

- Line 197: Please re-write

Response: Done. Now lines 222-225.

“For this survey, 20 expert participants were selected from the academy, the Undersecretariat for Fisheries and Aquaculture (SUBPESCA), National Fisheries and Aquaculture Service (SERNAPESCA), Fisheries Development Institute (IFOP), and specialist consultants’ staff”.

- Several of the attributes assessed and presented in table S3 are group level attributes. Why did you assessed these at the individual level, while the response is a group level variable (e.g. abundance of target species, fleet size)

Response: Thank you for highlighting this. We want to have a global vision about MEABRs, so we want to have individual and group answers. Moreover, the supplementary information S3 Table was modified, by adding an additional information about the sources (primary and secondary).

- Lines 225-233: it is still unclear why you did this

Response: We made this in order to identify strata (groups). It has been clarified in the supplementary material (see S1 Text).

- Lines 234-238: Authors do no explain why they chose to divide fishers into these groups and why is it interesting to assess these (e.g. why would you expect differences between those above and below 50 years old?). The way authors go about their methodology seems arbitrary and not theoretically informed, which makes it very challenging to follow what they are trying to do.

Response: Thank you for highlighting this. In lines 261-269, we have added more references and considered only three important factors, according to the objectives of this study.

“In order to analyze differences between important factors which are fundamental for the collective action, surveillance and logistical aspects in MEABRs, we classified the survey respondents into three main factors: i) gender (male or female) to assess the participation of women within the organization (57,62,73,96), ii) fishers’ organizational roles (leader or member), their participation into organizations of which they are part (97,98), and iii) MEABR accessibility or distance from fishing coves ‘caletas’ to the MEABR (accessible, less than 0.5 km or less accessible, more than 0.5 km), suggesting that human disturbances of many species and ecosystems are stronger in areas with higher physical accessibility (99–101)”.

Results

- Line 181: Re-write subtitle as it’s not very clear

Response: Done. In new line 317, we have changed the subtitle to: “Perceptions on gender, organizational role, and accessibility”.

- Lines 282-286: this should go in methods

Response: Done. We have moved this part to methods (now lines 274-278).

- Lines 362-363: Can you provide references for this? Authors state this as obvious, but it is not entirely clear to me if this is so (and I think by manage they mean enforce)

Response: In new lines 392-398, we have explained more details and included references.

“The accessibility of the MEABRs (measured as distance) can be related to the threat of poaching, i.e. in more distant MEABRs it is more difficult to enforce control, according to several authors (58,67,105). For the more accessible MEABRs only four attributes were more sensitive among the six dimensions described (i.e. ‘Other_Sp’, ‘Abund_Level’, ‘Mitigation’ and ‘Sis_Survei’). Regarding the technological dimension, the presence of surveillance (‘Sis_Survei’) was considered most influential in the more accessible MEABRs (<0.5km)”.

- Line 363-364: phrase is incomplete

Response: In new lines 394-396, the phrase was completed.

“For the more accessible MEABRs only four attributes were more sensitive among the six dimensions described (i.e. ‘Other_Sp’, ‘Abund_Level’, ‘Mitigation’ and ‘Sis_Survei’)”.

Discussion

- Line 384-385: It is not entirely clear where this statement is coming from

Response: In new lines 416-417 this statement has been referenced.

“In general, the MEABR system has continually evolved and adapted to a new socio-ecological reality (71)”.

- Line 386-388: This is a key statement, but should go in the introduction and better explained and referenced

Response: Thank you for this comment which indeed is an important one. Now lines 115-122 in the new introduction, we explained better and added references.

“Indeed, the perceptions of stakeholders (including fishers) are important indicators of the performance of the MEABR system, particularly when governance is deficient (76,77). Thus, for many of the world’s fisheries, including the Chilean exclusive access regime, a more holistic evaluation of the ecological and human dimensions is necessary (27,64,78–82). However, as far as we know, few studies in the region have systematically documented the perceptions of fishers in a socio-ecological context. Thus “each coastal territory and the fishers who live in it are heterogeneous, consequently although changes may be seen as transverse, no generalizations can be made regarding the local responses” ((73) page. 177)”.

- Gender perspective. This is a very interesting section, but requires a better theoretical grounding in the introduction so that readers understand from the beginning that assessing this was an objective of the study

Response: We thank the reviewer for highlighting this. In the new introduction, we incorporated theoretical grounding about this gender perspective (now lines 107-114).

“One recent work (71) concluded that if fishers’ organizations and consultants take as little part in the collective decision-making process as they have so far, it is unlikely that the necessary adjustments will be made to the system. Indeed, the different viewpoints of more men and women regarding management should be taken into account, in order to obtain a better understanding of the social interaction of women in an activity traditionally considered to be male-dominated (57,72–74). That's why women also need to have an increased right to participate in decision-making processes (75)”.

- Position perspective. It is not entirely clear what do authors refer to by “position perspective”. Is it the role MEARBs play in their livelihood?

Response: In new line 470, we have replaced “position perspective” by “organizational roles perspective”, which is related to the fisher role within the fishers’ organizations, such as leader or member.

- Line 459: please provide references, there are several studies looking at this, specifically for MEARBs in Chile. It would be helpful if you could expand on this, as it is a critical management issue

Response: In new lines 491-496, we have provided references about poaching.

“Poaching is one of the main factors influencing the scarcity of the target resource and one of the most critical problem perceived by fishers (67,69,71,87,112,113). Thus, ignoring illegal activity results in false conclusions (over- and under-representation) about the state and trends of fisheries; it also adds to the increased costs associated with surveillance and reduced income for fishers (68). Consequently, this leads to conflicts among members, with some leaving the association altogether”.

- Line 475: This line starts with a reference, which is unclear

Response: In new lines 510-511, we rephrased the paragraph.

“In order to promote the continuation of artisanal fishing within the coastal management plans, interaction among all stakeholders needs to be improved (114)”.

- Line 485-489: Authors could also look at the work by Cinner and others (e.g. Disentangling the complex roles of markets on coral reefs in northwest Madagascar) to complement this (distance to market). But it is also important to consider that those MEARB that are more accessible are easier to enforce, so there is no simple explanation for this distance/protection relationship

Response: Thank you for this comment which is indeed an important. We have added new information provided to improve the overall quality of our review. In new lines 526-532, we added the reference of Cinner et al. (99) and Maire et al. (115).

“We can complement this topic from the findings of Cinner et al. (99) and Maire et al. (115), who showed that reef fish biomass is strongly related to the accessibility of both markets and local communities. This means that market proximity affects fishing gear (technique effect), wealth, and selling strategies (scale effect) of coastal communities. In our case, we considered that other drivers could influence the performance of the MEABRs due to their variability and the effect of open access areas in the region, which are under-researched in our study zone”.

Conclusion

- Lines 508-512: I am not entirely sure the data supports this hypothesis. Certainly, the first section, but not the willingness to use the resource more sustainably

Response: In new lines 549-552, we have clarified this conclusion.

“This study shows that the perceptions of different stakeholders about the MEABR system influence their local fisheries management expectations in Biobio Region. We observed that individuals with differing gender and organizational role, and the accessibility of the MEABR influenced the expectations of this current system”.

- Line 526-228: Very unclear. Why are these organisations characterized as fragile and dependent? What coaching do you refer to?

Response:

For better understanding, we modified the expression **“fragile”** to **“weak”**: “Our results showed limited communication and relationships between fishers and other stakeholders, which makes fisher’s organizations weak and dependent on external sources” (now lines 565-567).

For better understanding, we modified the expressions **“advise and coach”** to **“enhance and increase”**: “Therefore, government managers and decision-makers should enhance and increase relational capacities (within organizations, among organizations and between organizations and the local environment) to improve their social capital on a more permanent basis as viable alternatives to their current modes of fishing” (now lines 567-571).

In new lines 563-574, we have clarified this in the conclusion.

“The ecological and social dimensions were most influential in shaping the fishers’ perceptions of successful MEABRs. Based on this, there is a dynamic exchange between these approaches and their complexity in the system. Our results showed limited communication and relationships between fishers and other stakeholders, which makes fisher’s organizations weak and dependent on external sources. Therefore, government managers and decision-makers should enhance and increase relational capacities (within organizations, among organizations and between organizations and the local environment) to improve their social capital on a more permanent basis as viable alternatives to their current modes of fishing. Besides, in the future, the government must increasingly include the fishers’ local knowledge and management strategies to ensure the best local understanding. Only a strong fisher-government partnership would help solve weaknesses in the system, promote continuity and facilitate responsible fisheries in MEABRs”.

- Line 528-530: I am not entirely sure how this comes out of the results, please clarify

Response: In the previous response we explained our results about communication and relationships between fishers and other stakeholders (now lines 565-574).

Examples of lines that need re-writing

42-43

Response: Now lines 45-48, we rewritten the paragraph #1 of the new introduction.

“However it is still necessary to improve the performance of fisheries where there are serious problems of overfishing and the management system is deficient (2). For this, several authors have suggested that integral monitoring should be improved for all fisheries, including artisanal fisheries (3,4)”.

67-68

Response: Now lines 68-72, we rewritten the paragraph #4 of the new introduction.

“Given this reality, various studies have shown that one of the strategies for improving management and sustainability of coastal fisheries is the Territorial Use Rights for Fisheries or TURF system (29–35). The TURFs are designed to assign exclusive rights and privileges to certain user groups in specific areas to ensure the rational use of resources (36–38)”.

73-74

Response: Now lines 79-83, we rewritten the paragraph #5 of the new introduction.

“The MEABRs gained traction in 1990 and over the years have been applied in a considerable geographic area. Despite some positive results both for environmental sustainability (41–43) and socio-economic effects (42,44–50), their management is still bedeviled by inefficiencies, because once a right is established, it is difficult to reverse or change it, even if unwanted results occur (48,51,52)”.

191-192

Response: Now lines 225-227, we rewritten as “Semi-structured interviews were conducted with the representatives of each institution. In addition, the questionnaire was applied to collect data about their perceptions and their experiences”.
