# Supplementary material for: Integrating human and ecological dimensions: The importance of stakeholders’ perceptions and participation on the performance of fisheries co-management in Chile
Source: PLoS One. 2021 Aug 11;16(8):e0254727. doi: 10.1371/journal.pone.0254727 (PMC8357100; doi:10.1371/journal.pone.0254727)
Supplement: Attachment — Submitted filename: Response to Reviewers.docx [file pone.0254727.s014.docx]

**Editor-in-Chief,**

*Journal of PLOS ONE*

RE: PLOS ONE Decision: Revision required [PONE-D-20-30809R1] -

[EMID:9e13eb0ce426fa07]

Below, we respond (in blue text) to the editor’s and reviewer’s comments.

Original comments from the Editor

Thank you for submitting your manuscript to PLOS ONE. After careful consideration, we feel that it has merit but does not fully meet PLOS ONE’s publication criteria as it currently stands. Therefore, we invite you to submit a revised version of the manuscript that addresses the points raised during the review process.

In your revision please address all the remaining comments suggested by reviewer #1, that also in my opinion will add clarity to the manuscript.

Authors’ response to the original comment from the Commissioning Editor

We would like to thank the Editor-in-Chief and the reviewer #1 for providing critical comments of our research. We have addressed all the points below. We provide clarification on the introduction section in the revised manuscript.

We consider that our revised manuscript has been greatly improved in terms of overall quality, adequately responding to all Editor and Reviewer concerns. Please find our detailed replies to reviewer below.

Reviewers’ comments

Reviewer #1

I congratulate authors for this new version of the manuscript. It has improved considerably. However, I still think there are some issues that need a bit of work. Nothing substantial, but small things that would certainly improve the quality of your work, especially in the intro.

Response: We thank the positive comments. Below, we have addressed the concerns mentioned.

- Paragraph 68-78 could be improved for clarity so that the original objectives of the AMERB system, and its current challenges are better differentiated. I would also recommend to comment on this work:

https://www.ingentaconnect.com/content/umrsmas/bullmar/2017/00000093/00000001/art00006 and https://conbio.onlinelibrary.wiley.com/doi/full/10.1111/conl.12637

Also, on this paragraph, the "this is the main problem" is not clear. Which one is the main problem? This paragraph still needs a bit of work, because is pivotal in your study, so needs to be crystal clear.

Response: We have modified some lines of the paragraph and hope that this is now clearer in the introduction. We also have considered the references cited by yourself (now lines 68-85).

“Given this reality, various studies have shown that one of the strategies for improving management and sustainability of coastal fisheries is the Territorial Use Rights for Fisheries or TURF system (29–35). The TURFs, also popularly known in Chile as the Management and Exploitation Areas for Benthic Resources-MEABR system (or AMERB, in its Spanish acronym), are designed to assign exclusive rights to artisanal fisher organizations in specific areas to harvest sustainably benthic species (36–38). Fishers must provide baseline information and a management plan to target a single or groups of benthic species. In addition, private consultants hired by each fisher organization assess the standing stock biomass of the target species through field surveys to set an annual harvest quota authorized by SUBPESCA. The objectives of MEABR are a governance transformation through co-management and to enhance the sustainability of small-scale fisheries (39). Thus, the TURF system, is a better administrative mechanism for controlling open-access resources, thus achieving an equitable distribution of benefits and maintaining social cohesion (29,40–42). Unfortunately, some TURFs have never produced the outcomes expected for various reasons due to scarce administrative, human and financial resources for surveillance of illegal fishing, among others (43,44). In this context, the main problem affecting the TURF system in Chile is the lack of a transdisciplinary approach that merges the ecological and social systems (45).”

- Paragraph 79-92 needs some editing. First, the MEARB system was conceptualized and some pilots started in 1990. It didn't gain traction until later. I also think you could be a more nuanced about the benefits/limitations and the overall performance of the system (see references above), as there are lots of successful cases. Now it reads as if the benefits are the exception.

Response: Thank for your observation, we have re-write and added some lines to contextualize the paragraph (now lines 86-100). We have changed this to:

“The MEABRs gained traction by the end-1990 and have had a considerable territorial impact over the years. In sites where MEABRs have been well applied, the density and size of target species increased substantially relative to open-access areas (46–53), generating positive outcomes for environmental sustainability (52,54,55) and socio-economic (42,50,55–60). However, their management is still bedeviled by inefficiencies, because once a right is established, it is difficult to reverse or change it, even if unwanted outcomes occur (59,61,62). For example, some MEARBs managing surf clams (63,64) could not solve the problems caused by the high temporal variability of this resource. As a result, the governance was abandoned and did not recover even when the resource itself recovered (59,61,65–70). Nevertheless, one of the favorable effects of the MEABRs has been the empowerment of fishers’ organizations, which have promoted collective actions among users (65,71). Collective action is broadly understood as “people joining together purposively for a common cause” (72). Also, it could be confrontational, depending on the relationship among stakeholders (73). Since their inception, MEABRs have been adapted to new regulations and fishing practices (74).”

-Paragraph 93-103 I think you mean "have not been met" in the second line. I would also say outcomes instead of results in the manuscript. The last sentences is a bit confusing (needs re-writing) as you jump quite unexpectedly from the local to worldwide.

Response: We thank the reviewer for highlighting these words, which were replaced. Furthermore, we have modified the last sentences for better understanding (now lines 101-113).

“The Biobio region in south-central Chile, is an example of how most of the outcomes envisaged for the MEABR system have not been met (75). In many cases, this lack of positive outcomes has in many cases caused frustration and the abandonment of MEABRs administered by artisanal fishers’ organizations (76–78). Management of the MEABRs is complex and fishers’ responses can have unforeseen consequences leading to failure. As stated earlier, many examples of the MEABR system are being applied with no consideration of the socio-ecological systems upon which they were implemented (59,61,65,79). The lessons learned with the MEABR system in Chile could be helpful for the TURF system worldwide. For example, finding out why the regulations have not been followed or respected could help to avoid bad outcomes the system has generated so far. And could explain why the lack governance that prioritizes participatory management has contributed to the overexploitation and poor performance of small-scale marine fisheries (80).”

Paragraph 115-122 the reference provided above does exactly that systematic documentation, so would be good if you could comment on that

- I think that there still isn't a proper section of the intro that talks about gender. There's a lot of literature on this which the authors could look at, which consider the different types or roles women have played, how those roles have been neglected and pathways to their inclusion. This becomes very relevant in the face of the newly approved law in Chile on gender in the fisheries sector, so I would strongly recommend authors to go a bit deeper here.

Response: Thanks for the valuable comment. We have added the following new paragraph (now lines 124-140) to contextualize and a bit deeper more about gender role in the fisheries sector

“In Latin America, women play a fundamental role in fishing-related activities (85,86). Despite this, women are not an entirely homogeneous category of people in the sector, rarely found in statistics or documented in socioeconomic evaluations (87,88). Therefore women are subject to various degrees of inequalities in access to and control over productive resources, services, employment opportunities, and empowered participation in decision-making (89,90). They have been made invisible for many years. However, in the last few decades, the relevant role of women in contributing to new forms of sustainability through their work and traditional knowledge has been emphasized (83). They gain more and more momentum and recognition in different instances and even as good managers and negotiators in the value chain and fishing system (67,91–93). Thus, in this changing scenario, it becomes relevant that gender mainstreaming should be an integral part of all small-scale fisheries development strategies (94). Moreover, that leads to implementing new regulations on gender equity, such as modification of the current Law No. 18.892, General Law on Fisheries and Aquaculture in Chile (to be enacted), which seeks to incorporate gender quotas and opportunities to reach spaces for dialogue regarding fisheries management. That is why women’s perceptions should include in any study of fisheries co-management performance.”

-Lines 182-186 please explain what an ESBA proposal is

Response: We now explain the ESBA (now lines 208-214).

“Approved and awarded: those MEABRs requested by a fisher’s organization with the baseline study (or ESBA, in its Spanish acronym, which describes the initial status of MEABR as for the conservation status of species, ecosystem, socio-economic condition, bathymetry, among other aspects); Approved but not awarded: those MEABRs that are available to be used but to date no organization is interested, has expired or has been returned by a fisher’s organization, or is pending a decision by competition among two or more organizations, Rejected: those MEABRs that did not get approval to be used because the process stopped earlier (Chavez et al. (110)).”
